# Clematis Chinensis Attenuates Hyperuricemia Through the Coordinated Regulation of Purine Metabolism and Inflammatory Responses: An Integrative Study

**DOI:** 10.3390/ph19060830

**Published:** 2026-05-26

**Authors:** Ze Fu, Hao Ju, Zi-Hao Chen, Yan-Chao Wu, Hui-Jing Li

**Affiliations:** Weihai Marine Organism and Medical Technology Research Institute, School of Marine Science and Technology, Harbin Institute of Technology, Weihai 264200, China; fz_apply2022@163.com (Z.F.); juhao17@mails.ucas.ac.cn (H.J.); czhowu@163.com (Z.-H.C.); yanchaowu@hit.edu.cn (Y.-C.W.)

**Keywords:** hyperuricemia, *Clematis chinensis* Osbeck, xanthine oxidase, renal injury, inflammation, urate transport

## Abstract

**Background/Objectives**: Hyperuricemia is a metabolic disorder characterized by renal dysfunction and systemic inflammation. While *Clematis chinensis* Osbeck is traditionally used for gout-related conditions, its chemical basis and precise mechanisms remain poorly understood. This study aimed to characterize the bioactive fraction (CWE-60EF) and elucidate its multi-target regulatory mechanisms against hyperuricemia. **Methods**: Qualitative and quantitative chemical profiling of CWE-60EF was performed using high-resolution LC-MS/MS. Its anti-hyperuricemic activity was validated using in vitro xanthine oxidase (XOD) inhibition, a zebrafish model, and HK-2 cell injury models. Mechanisms were explored through an integrated approach combining bioinformatics, Mendelian randomization (MR), and molecular docking. **Results**: A total of 50 compounds, primarily alkaloids and flavonoids (e.g., magnoflorine and phloretin), were characterized in CWE-60EF, and major marker compounds were quantitatively standardized. The fraction significantly inhibited XOD activity and rescued hyperuricemia-associated phenotypes in zebrafish. In HK-2 cells, CWE-60EF suppressed adenosine- and urate-induced cellular injury and the transcriptional expression of pro-inflammatory cytokines (IL-6 and IL-1*β*). MR analysis provided genetic evidence supporting IL-6 as a causal mediator of gout risk. Integrative analysis revealed that the protective effects of CWE-60EF are mediated through the coordinated regulation of purine metabolism, inflammatory cascades, and urate transporters (URAT1/GLUT9). **Conclusions**: This study demonstrates that CWE-60EF is a quantitatively standardized bioactive fraction that exerts anti-hyperuricemic, renoprotective, and anti-inflammatory effects by modulating uric acid metabolism and inflammation. By integrating genetic causality with phytochemical validation, our findings provide a novel mechanistic foundation for the traditional application of *C. chinensis* in hyperuricemic disorders.

## 1. Introduction

Hyperuricemia (HUA), defined by elevated serum uric acid (UA) levels, has become increasingly prevalent worldwide and represents a growing public health concern [1,2]. In addition to being the primary biochemical basis of gout, HUA is associated with an increased risk of chronic kidney disease (CKD), cardiovascular disorders, and metabolic syndrome [3,4,5]. Recent studies highlight a bidirectional relationship between hyperuricemia and renal dysfunction: elevated UA impairs kidney function through mechanisms such as oxidative stress and inflammation, while reduced renal excretion further aggravates urate accumulation. Therefore, therapeutic strategies that provide concurrent urate-lowering effects and renal protection are of significant clinical interest [6].

Current urate-lowering therapies (ULTs) primarily focus on either inhibiting UA production (e.g., allopurinol and febuxostat) or promoting renal excretion (e.g., benzbromarone) [7,8]. Although some clinical agents exhibit pleiotropic antioxidant or anti-inflammatory properties, their primary efficacy often targets a predominant metabolic pathway. Furthermore, the clinical utility of standard ULTs can be limited by adverse effects, such as hypersensitivity and cardiovascular safety concerns [9,10]. This underscores the need for multi-target therapeutic approaches that can simultaneously address the complex metabolic and inflammatory alterations associated with HUA—a knowledge gap that requires the integration of causal genetic evidence with phytochemical validation.

Recent evidence reveals that HUA-related renal injury is mediated by intracellular metabolic disturbances, mitochondrial dysfunction, and inflammatory activation in renal tubular epithelial cells [11,12]. Central to these processes are nuclear receptors such as peroxisome proliferator-activated receptors (PPARs), which serve as pivotal nodes coordinating lipid metabolism, inflammatory gene transcription, and the expression of urate transporters [13,14,15]. While the involvement of PPAR signaling in metabolic homeostasis is well-documented, its specific role as a bridging mediator between renal inflammation and urate transport in hyperuricemia remains to be fully elucidated.

Natural products and Traditional Chinese Medicine (TCM), characterized by their multi-component modes of action, are promising candidates for such integrated therapeutic strategies [16,17,18]. However, the relationships between active phytochemicals, molecular targets, and disease phenotypes remain incompletely understood, particularly regarding the genetic necessity of these targets in humans. *Clematis chinensis* Osbeck has long been used in traditional medicine for rheumatic and “bi-syndrome” (gout-associated) conditions. Previous phytochemical studies have identified constituents like alkaloids, triterpenoids, and flavonoids with broad anti-inflammatory activities [19,20,21]. While these traditional claims are extensive, modern experimental validation has often focused on crude extracts, and the molecular basis of standardized fractions, such as our previously identified CWE-60EF, has not been systematically investigated through a genomic lens.

In the present study, we employed an integrative strategy combining qualitative and quantitative LC-MS/MS analyses, transcriptomics, and Mendelian randomization to identify causal therapeutic targets. By bridging the gap between traditional use and evidence-based pharmacology, we evaluated whether the standardized constituents of CWE-60EF target the PPAR-mediated inflammatory axis to provide renoprotection. Our findings provide novel mechanistic support for the clinical application of *C. chinensis* in gout-related disorders.

## 2. Results

### 2.1. Bio-Guided Fractionation and Initial Screening of Anti-Hyperuricemic Activity

**Figure 1:**
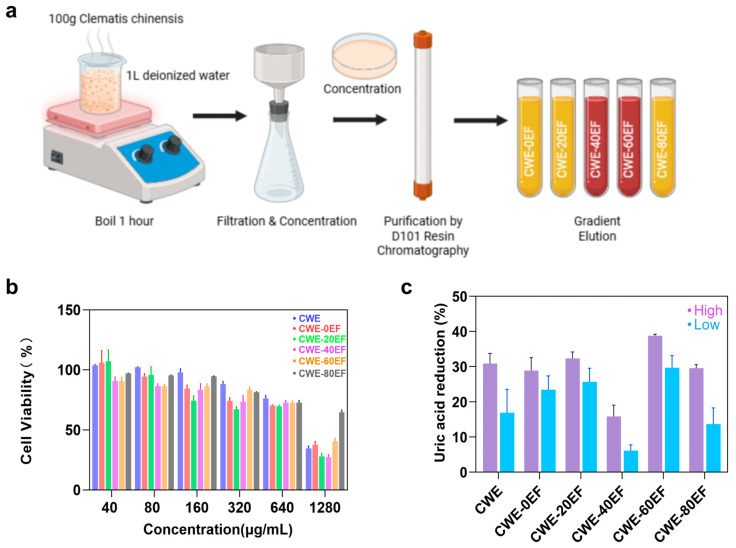
Preparation of CWE-60EF and evaluation of its anti-hyperuricemic activity. (**a**) Schematic workflow of the bio-guided fractionation of Clematis chinensis extracts (CWE) using D101 macroporous resin with gradient elution, yielding five fractions (CWE-0EF to CWE-80EF); (**b**) Cytotoxicity of different fractions in HK-2 cells. Cell viability was determined by the MTT assay after 24 h of treatment at concentrations ranging from 40 to 1280 μg/mL; (**c**) Effects of different fractions on uric acid (UA) levels in an adenosine-induced HK-2 cell model. Low (80 μg/mL) and high (320 μg/mL) concentrations were used to evaluate UA-lowering activity. Data are presented as mean ± SD (*n* ≥ 3).

To identify fractions with potential anti-hyperuricemic activity, *C. chinensis* was sequentially fractionated using D101 macroporous resin with gradient elution, yielding five fractions ranging from CWE-0EF to CWE-80EF (Figure 1a).

#### 2.1.1. Cytotoxicity Assessment and Dose Optimization

The cytotoxicity of each fraction was evaluated in HK-2 cells using the MTT assay. As shown in Figure 1b, all fractions showed low cytotoxicity at concentrations of 40–320 μg/mL, with cell viability remaining above 85%. At 640 μg/mL, most fractions still maintained approximately 70% cell viability. Based on these results, 320 μg/mL was selected as the upper limit to maximize the potential metabolic regulatory effects while ensuring that the cells remained in a healthy physiological state (viability > 85%).

#### 2.1.2. UA-Lowering Effects in Adenosine-Induced HK-2 Cells

An adenosine-induced HK-2 cell model was used to evaluate the anti-hyperuricemic effects of the fractions by measuring intracellular uric acid (UA) levels (Figure 1c). Among the tested fractions, CWE-60EF exhibited the most robust anti-hyperuricemic capacity, reducing intracellular UA levels by 41.2% ± 3.5% (*p* < 0.001), which significantly outperformed the crude extract (25.3%) and other fractions (Figure 1c). This quantitative superiority demonstrated that the 60% ethanol eluate concentrated the primary active constituents responsible for the renal metabolic regulation of *C. chinensis*.

### 2.2. Systematic Chemical Characterization and Target Prediction of CWE-60EF

To clarify the chemical basis of the anti-hyperuricemic activity, CWE-60EF was analyzed by UPLC-QE-Orbitrap-MS/MS in both positive and negative ion modes.

#### 2.2.1. Qualitative Profiling of Chemical Constituents

**Figure 2:**
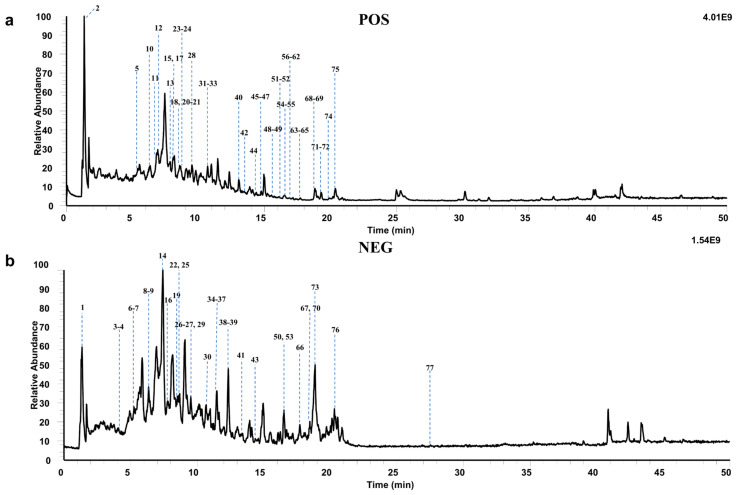
Qualitative chemical characterization of CWE-60EF by HR–LC–MS/MS analysis. (**a**) Total ion chromatogram (TIC) of CWE-60EF acquired in positive ionization mode; (**b**) Total ion chromatogram (TIC) of CWE-60EF acquired in negative ionization mode. Individual peaks are numbered according to the identified compounds listed in Supplementary Table S3. The HR–MS profiles provided the basis for the annotation of major constituents, including magnoflorine, phloretin, and phenolic acid derivatives.

The TICs showed good chromatographic resolution and peak capacity (Figure 2a,b). A total of 77 characteristic peaks were detected, and 50 compounds were confidently identified or tentatively annotated based on accurate mass data, MS/MS fragmentation patterns, and database matching (Supplementary Table S3; Supplementary Note S4). These compounds were mainly classified as alkaloids, phenolic acids, flavonoids, and triterpenoids. Representative compounds such as magnoflorine, clematichinenoside A, and isochlorogenic acid C were further supported by characteristic fragmentation patterns (Supplementary Figure S2).

#### 2.2.2. Target Prediction and Compound–Target Network Construction

Putative targets of the identified compounds were predicted using SwissTargetPrediction and SEA. After probability-based filtering, 598 candidate targets were obtained. These targets were then intersected with hyperuricemia-related genes to obtain disease-relevant candidate targets. This analysis provided a preliminary basis for subsequent network-based target prioritization. By systematically mapping the chemical space onto the disease target space, this analysis established a preliminary link between the diverse constituents of CWE-60EF and hyperuricemia-related pathology.

To evaluate chemical standardization, we quantified 7 major marker compounds in the CWE-60EF fraction across three independent batches. As shown in Table 1, Magnoflorine (34.93 ± 14.39 μg/mL) and Phloretin (14.51 ± 1.40 μg/mL) were identified as the primary active constituents. The triterpenoids (oleanolic, ursolic, corosolic, and glycyrrhetinic acids) were present at concentrations below 1 μg/mL, while Esculin was not detected in this specific fraction. The high consistency of these profiles, further supported by the TICs in Supplementary Figure S3, confirms the reproducibility of our fractionation and extraction methodology.

### 2.3. Integrative Bioinformatics Analysis Identified IL6 as a Key Hub Target of CWE-60EF

#### 2.3.1. Identification of Gout-Associated Gene Modules by WGCNA and DEG Analysis

**Figure 3:**
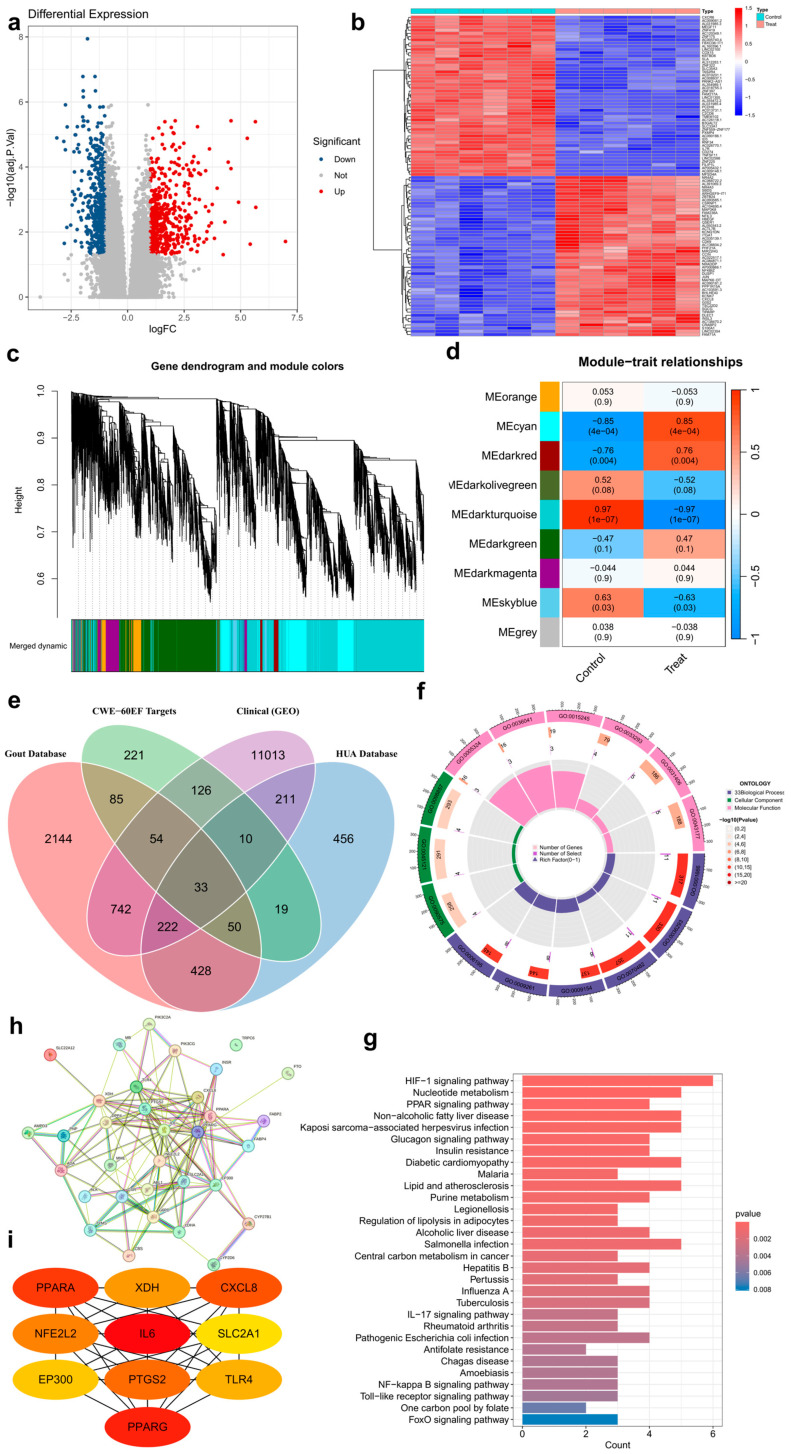
Integrated bioinformatics analysis of core targets and enriched pathways. (**a**,**b**) Volcano plot and heatmap of differentially expressed genes (DEGs) in the GSE160170 dataset; (**c**,**d**) WGCNA showing the gene dendrogram and module–trait relationship heatmap; the MEdarkturquoise module showed the strongest association with gout; (**e**) Venn diagram identifying 33 core targets by integrating DEGs, WGCNA module genes, predicted CWE-60EF targets, and gout-related targets; (**f**,**g**) GO and KEGG enrichment analyses of the 33 core targets; (**h**,**i**) Protein–protein interaction (PPI) network and hub gene ranking by CytoHubba. Nodes are colored from red to yellow according to degree score, and IL6 was identified as the top hub gene.

Transcriptomic analysis of the GSE160170 dataset identified DEGs between gout and control samples (Figure 3a,b). WGCNA identified several co-expression modules, among which the MEdarkturquoise module showed the strongest correlation with gout (r = 0.97, *p* = 1 × 10^−7^; Figure 3c,d).

#### 2.3.2. Identification of Core Targets Using an Integrated Screening Strategy

To streamline the identification of high-confidence targets, we utilized a tripartite screening strategy: intersecting transcriptomic signatures from clinical gout samples (DEG/WGCNA) with the predicted botanical targets and established disease genes (Figure 3e). This analysis identified 33 overlapping genes as core targets of CWE-60EF against gout. GO enrichment analysis (Figure 3f) showed that these genes were mainly associated with transmembrane transport, metabolic regulation, transporter activity, substrate binding, and membrane-related cellular components. KEGG pathway analysis (Figure 3g) indicated enrichment in the HIF-1 signaling pathway, nucleotide metabolism, the PPAR signaling pathway, insulin resistance, and inflammatory pathways, including NF-kappa B, Toll-like receptor, and IL-17 signaling.

#### 2.3.3. PPI Network Construction and Identification of IL6 as the Hub Gene

A PPI network was constructed for the 33 core targets (Figure 3h). Hub genes were ranked using the Degree algorithm in CytoHubba plugin (version 0.1). IL6 had the highest degree score and was identified as the key hub gene (Figure 3i). While IL-6 exhibited the highest degree of network centrality, its biological relevance and causal role in gout development were further investigated in the subsequent MR and experimental validations to avoid overinterpreting its computational importance.

#### 2.3.4. Molecular Docking and Molecular Dynamics Simulations Supported the Stable Binding of CWE-60EF Constituents to XDH

**Figure 4:**
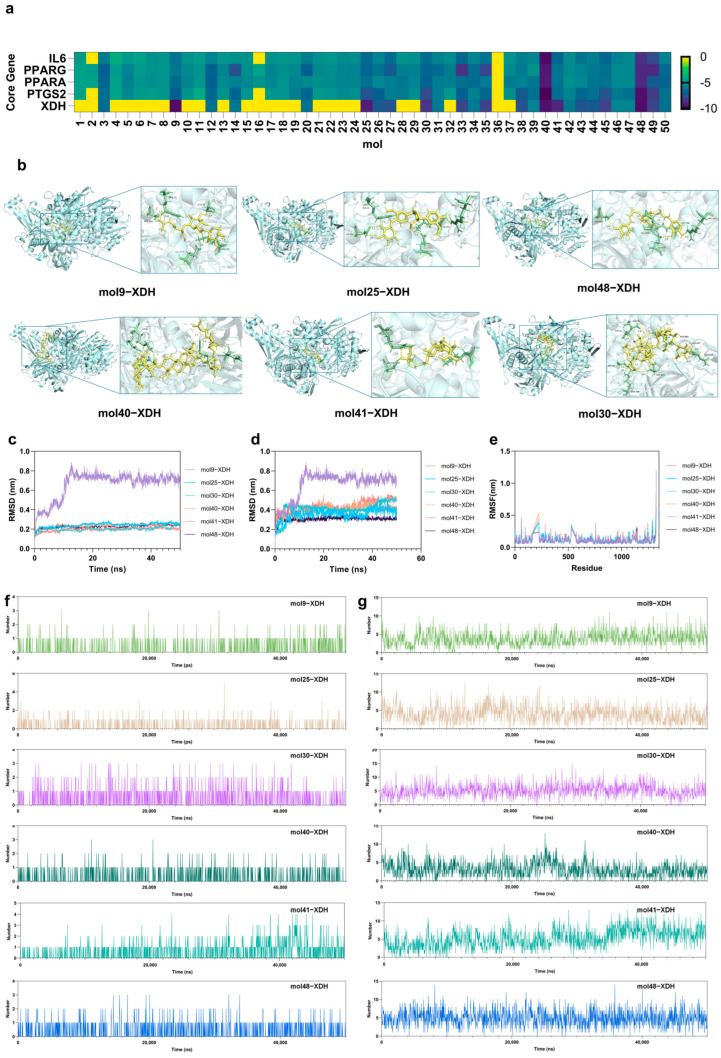
Molecular docking and molecular dynamics analysis of CWE-60EF constituents with representative hub targets. (**a**) Heatmap of docking scores for the identified compounds against the selected hub targets (In panel (**a**), the color transition from yellow to purple represents the binding affinity scores ranging from 0 to −10 kcal/mol, where purple indicates stronger binding interaction); (**b**) Representative three-dimensional binding modes of the six selected ligands (mol9, mol25, mol30, mol40, mol41, and mol48) in the active pocket of XDH; (**c**) Root mean square deviation (RMSD) trajectories of the XDH backbone during molecular dynamics (MD) simulations; (**d**) RMSD trajectories of the six ligands during the 50 ns MD simulations; (**e**) Root mean square fluctuation (RMSF) of XDH residues during the MD simulations; (**f**) Changes in the number of hydrogen bonds formed between the ligands and XDH during the MD simulations; (**g**) Distribution of hydrogen bond distances in the ligand-XDH complexes. A distance of 0.35 nm was used as the cutoff for hydrogen bond analysis.

To further explore the binding basis of CWE-60EF, molecular docking was performed between the 50 identified constituents and five hub targets, including IL6, PPARA, PPARG, XDH, and PTGS2 (Figure 4a). Based on docking scores, six compounds with the lowest binding energies were selected for further analysis. Because XDH plays a key role in the final step of purine metabolism and showed strong inhibitory activity in the biochemical assay, it was selected for molecular dynamics (MD) simulation.

The docking results showed that these compounds occupied the active pocket of XDH and interacted with key residues, including ASP594, ARG32, LYS269, GLU679, and ARG233 (Figure 4b). Fifty-nanosecond MD simulations were then performed for the six XDH-ligand complexes using GROMACS. The RMSD values of both the protein backbone and ligands reached equilibrium early in the simulation and remained relatively stable throughout the 50 ns trajectory (Figure 4c,d). RMSF analysis showed limited fluctuations in most residues, including those near the catalytic region (Figure 4e). In addition, the number of hydrogen bonds remained stable, and bond distances were mainly distributed within 0.35 nm (Figure 4f,g). These results indicate stable binding between XDH and the selected CWE-60EF constituents. The stable binding energy and early equilibrium in MD trajectories suggest that these constituents may act as competitive inhibitors within the XDH active pocket, potentially translating into the functional inhibition of uric acid production observed in our biochemical assays.

### 2.4. Clinical Validation and Diagnostic Nomogram Construction

To evaluate the clinical relevance and diagnostic utility of the representative hub genes, their expression patterns and predictive performance were further assessed using the clinical dataset.

#### 2.4.1. Expression Validation of Hub Genes in Clinical Samples

**Figure 5:**
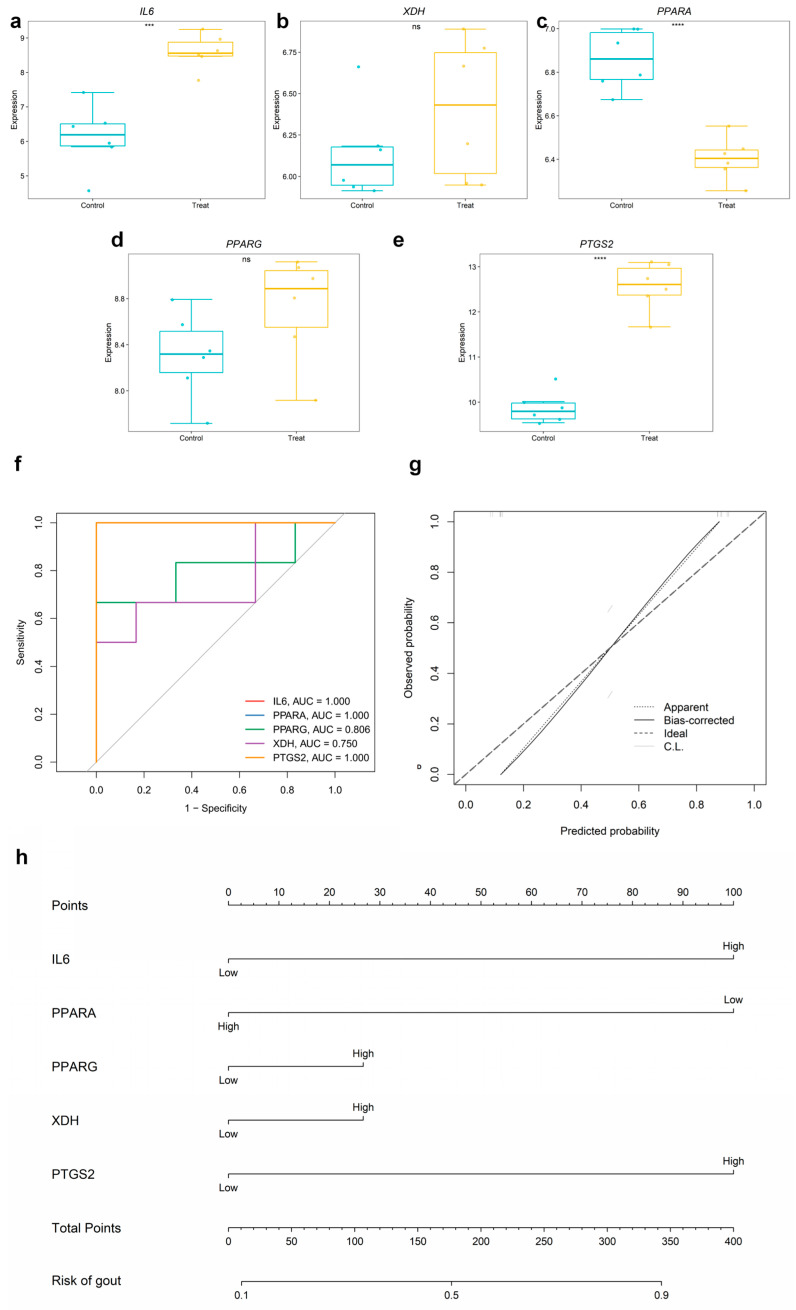
Clinical validation and diagnostic model construction based on representative hub genes. (**a**–**e**) Expression levels of IL6, XDH, PPARA, PPARG, and PTGS2 between Control and Treat groups. Individual data points are shown as dots. *** *p* < 0.001, **** *p* < 0.0001, and *ns* = not significant; (**f**) ROC curves of the five core genes, where the gray dashed line represents the reference (AUC = 0.5); (**g**) Calibration curve of the model; the dashed line represents the ideal prediction; (**h**) Nomogram for predicting the risk of gout.

Expression analysis in the clinical dataset showed that IL6 and PTGS2 were significantly upregulated in gout patients compared with controls, whereas PPARA was significantly downregulated. In contrast, PPARG and XDH showed an upward trend in gout samples, although the differences did not reach statistical significance (Figure 5a–e).

#### 2.4.2. Diagnostic Evaluation and Nomogram Construction

ROC curve analysis indicated that all five representative hub genes had diagnostic value for distinguishing gout from control samples. Among them, IL6, PPARA, and PTGS2 exhibited the strongest discriminatory performance, each with an AUC of 1.000, whereas PPARG and XDH yielded AUC values of 0.806 and 0.750, respectively (Figure 5f). However, it should be acknowledged that these perfect AUC values likely reflect the relatively small sample size of the clinical discovery dataset and may indicate unintended overfitting. Thus, these findings should be interpreted with caution and require validation in larger, independent cohorts. A diagnostic nomogram incorporating these representative hub genes was subsequently established for quantitative estimation of gout risk (Figure 5h). In the nomogram, IL6, PPARA, and PTGS2 contributed more prominently to the predictive model, whereas PPARG and XDH showed relatively smaller contributions. The calibration curve demonstrated good agreement between predicted and observed probabilities, indicating satisfactory calibration and reliability of the model (Figure 5g).

### 2.5. Genomic Causality and Immune Landscape Analysis of the IL6-Gout Axis

To further investigate the role of IL6 in gout, two-sample Mendelian randomization (2SMR) analysis and immune infiltration analysis were performed to evaluate its potential causal effect and immune associations.

#### 2.5.1. Genetic Evidence Supporting a Causal Association Between IL6 and Gout

As supportive genetic evidence for the target causal role of IL6 (rather than direct validation of CWE-60EF), a two-sample Mendelian randomization analysis was performed. Fifteen SNPs were utilized as instrumental variables, all of which exhibited sufficient strength with F-statistics ranging from 24.55 to 36.86 (Supplementary Table S1), effectively ruling out weak instrument bias.

**Figure 6:**
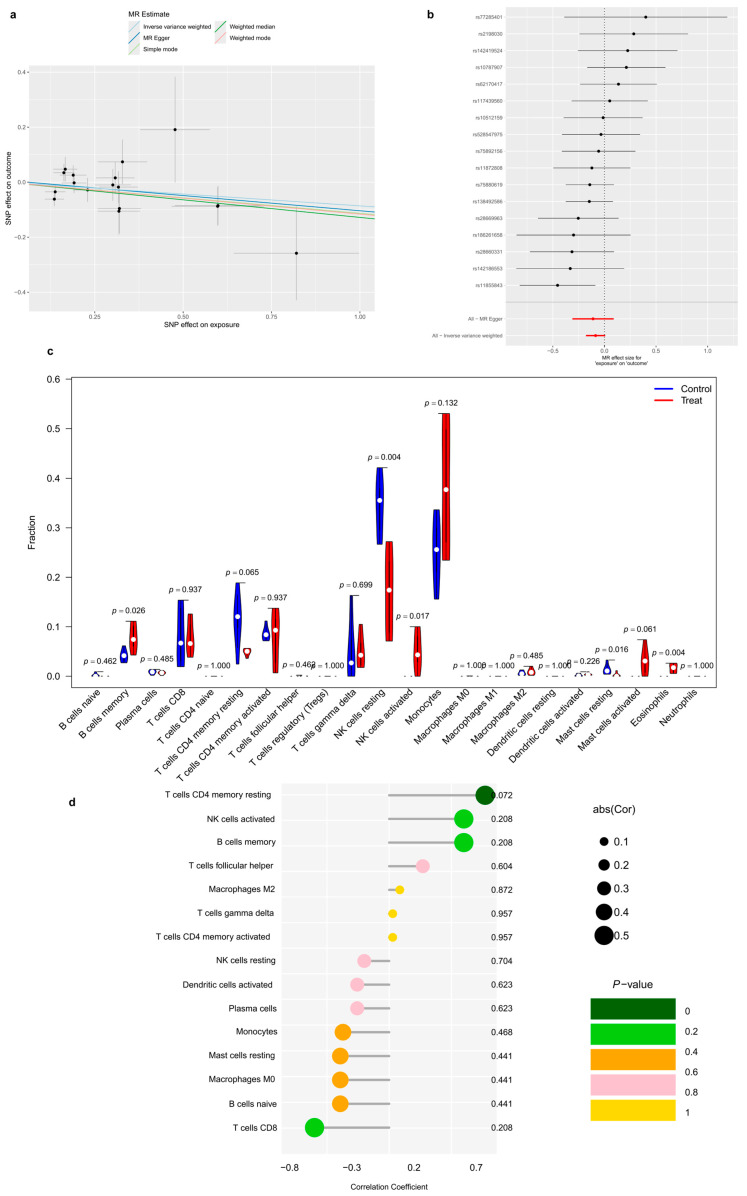
Mendelian randomization and immune infiltration analyses of the IL6-gout axis. (**a**) Scatter plot showing the estimated effect of IL6 exposure on gout risk. The slopes of the lines represent Mendelian randomization estimates obtained using different methods; (**b**) Forest plot showing the individual and combined effects of 15 single-nucleotide polymorphisms (SNPs) on gout risk. The red line represents the overall MR Egger estimate, and the gray horizontal lines represent the 95% confidence intervals (CIs) for each SNP; (**c**) Immune cell infiltration analysis using CIBERSORT. The violin plot compares the proportions of 22 immune cell types between the gout group (Treat) and healthy controls (Control). *p*-values are indicated above the violins; (**d**) Lollipop plot showing the correlations between IL6 expression and 22 immune cell types. Correlations were evaluated using Spearman’s rank correlation analysis. Dot size represents the absolute correlation coefficient (|r|), and dot color represents the p-value. Data in panel (**c**) are presented as mean ± SD.

As shown in Figure 6a, a suggestive positive association was observed across multiple MR models. The IVW method identified that genetically predicted circulating IL6 levels were associated with a modestly increased risk of gout (OR = 1.049, 95%CI: 1.002–1.098, *p* = 0.040; Figure 6b).

Sensitivity analyses further supported the robustness of this suggestive association. The MR-Egger intercept test showed no evidence of horizontal pleiotropy (*p* = 0.632), and Cochran’s Q test indicated no significant heterogeneity (*p* = 0.558). Given the small effect size and borderline statistical significance, these MR findings provide supportive genetic evidence that IL6 may act as a causal mediator in gout development, thereby justifying its selection as a therapeutic target for CWE-60EF.

#### 2.5.2. Immune Infiltration Characteristics in Gout

The CIBERSORT algorithm was used to estimate the proportions of 22 immune cell types in gout and control samples (Figure 6c). Compared with healthy controls, gout samples showed increased proportions of monocytes, resting NK cells, and gamma delta T cells, while CD8^+^ T cells and resting CD4^+^ memory T cells were reduced. These results indicate that gout is associated with altered immune cell composition.

#### 2.5.3. Association Between IL6 Expression and Immune Cell Infiltration

Correlation analysis between IL6 expression and immune cell infiltration was performed (Figure 6d). IL6 expression was positively correlated with resting CD4^+^ memory T cells and activated NK cells, whereas negative correlations were observed with naive B cells and M0 macrophages. These findings suggest that IL6 may be associated with immune remodeling in gout.

### 2.6. In Vivo Effects of CWE-60EF in Hyperuricemic Zebrafish

A zebrafish hyperuricemia (HUA) model was used to further evaluate the in vivo effects of CWE-60EF.

#### 2.6.1. Effects on Biochemical Indicators

**Figure 7:**
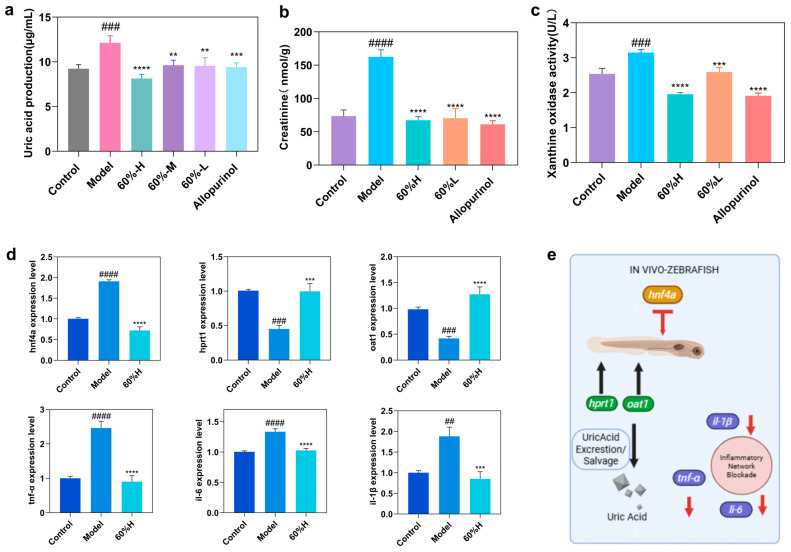
In vivo validation of CWE-60EF in a zebrafish model of hyperuricemia. (**a**–**c**) Quantitative analysis of uric acid (UA) production, creatinine (Cr) levels, and xanthine oxidase (XOD) activity in zebrafish; (**d**) RT-qPCR analysis of genes related to purine metabolism and urate transport, including *oat1*, *hnf4a*, and *hprt1*, as well as inflammatory cytokines, including *tnfa*, *il1b*, and *il6*; (**e**) Schematic summary of the in vivo effects of CWE-60EF on metabolic and inflammatory alterations in hyperuricemic zebrafish. In panel (**e**), black arrows indicate promotion or activation, the red T-bar indicates inhibition of protein expression, and red downward arrows indicate a decrease or suppression of inflammatory cytokine levels. Data are presented as mean ± SD, analyzed by one-way ANOVA followed by Tukey’s post hoc test. ## *p* < 0.01, ### *p* < 0.001, #### *p* < 0.0001 vs. the control group; ** *p* < 0.01, *** *p* < 0.001, **** *p* < 0.0001 vs. the model group.

Compared with the control group, the HUA model group showed significantly increased uric acid (UA) production, creatinine (Cr) levels, and xanthine oxidase (XOD) activity (Figure 7a–c). CWE-60EF treatment reduced these changes, and the high-dose group showed the strongest effect. UA production and XOD activity in the high-dose group were comparable to those in the allopurinol group.

#### 2.6.2. Regulation of Purine Metabolism, Urate Transport, and Inflammatory Genes

RT-qPCR analysis was performed to examine the expression of genes related to purine metabolism, urate transport, and inflammation in zebrafish (Figure 7d). Compared with the model group, CWE-60EF treatment decreased hnf4a, tnfa, il6, and il1b expression, while increasing hprt1 and oat1 expression. A schematic summary of these in vivo effects is shown in Figure 7e.

### 2.7. In Vitro Protective Effects of CWE-60EF in HK-2 Cells

To further evaluate the protective effects of CWE-60EF, two HK-2 cell models were used, including an adenosine-induced model and a uric acid (UA)-induced model.

#### 2.7.1. Effects of CWE-60EF in the Adenosine-Induced HK-2 Cell Model

**Figure 8:**
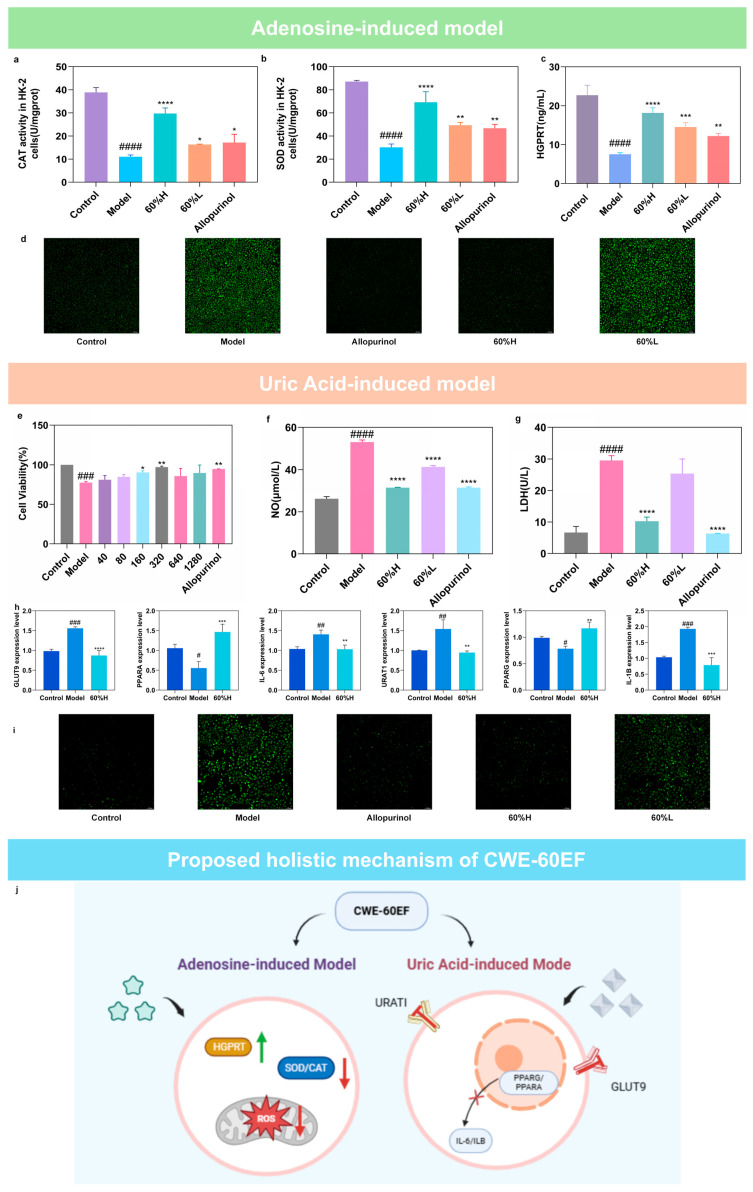
Protective effects of CWE-60EF in HK-2 cell models. (**a**–**d**) Adenosine-induced model: effects of CWE-60EF on (**a**) CAT activity, (b) SOD activity, (**c**) HGPRT levels, and (**d**) intracellular ROS levels detected by DCFH-DA staining. (**e**–**i**) UA-induced model: (**e**) effects of different concentrations of CWE-60EF on cell viability, (**f**) NO levels, (**g**) LDH release, (**h**) RT-qPCR analysis of SLC2A9 (GLUT9), SLC22A12 (URAT1), PPARA, PPARG, IL6, and IL1B expression, and (**i**) intracellular ROS levels detected by DCFH-DA staining. (**j**) Proposed schematic illustration of the protective effects of CWE-60EF on metabolic, oxidative, and inflammatory alterations in HK-2 cells. In panel (**j**), green upward arrows indicate upregulation or increased activity; red downward arrows indicate downregulation or decreased levels; and red T-bars indicate inhibition. Data are presented as mean ± SD. # *p* < 0.05, ## *p* < 0.01, ### *p* < 0.001, and #### *p* < 0.0001 vs. the control group; * *p* < 0.05, ** *p* < 0.01, *** *p* < 0.001, and **** *p* < 0.0001 vs. the model group.

In the adenosine-induced model, CWE-60EF increased CAT and SOD activities compared with the model group (Figure 8a,b). HGPRT expression, which was reduced in the model group, was also restored after treatment (Figure 8c). In addition, fluorescence imaging showed that intracellular ROS accumulation was decreased by CWE-60EF treatment (Figure 8d).

#### 2.7.2. Effects of CWE-60EF in the UA-Induced HK-2 Cell Model

In the UA-induced model, CWE-60EF partially restored cell viability after UA exposure (Figure 8e). UA-induced increases in NO production and LDH release were also reduced after treatment (Figure 8f,g). At the transcriptional level, CWE-60EF reversed the UA-induced upregulation of GLUT9, URAT1, IL6, and IL1B and partially restored PPARA and PPARG expression (Figure 8h). Fluorescence imaging further showed reduced intracellular ROS accumulation after CWE-60EF treatment (Figure 8i). A schematic summary of the proposed effects of CWE-60EF is shown in Figure 8j.

In summary, these in vivo and in vitro findings validated our earlier computational predictions, confirming that CWE-60EF targets the hub genes (e.g., IL6 and PPARs) identified through network pharmacology to exert its renoprotective and anti-inflammatory effects.

## 3. Discussion

### 3.1. Constituents of CWE-60EF and Potential Interaction with XDH

The biological activity of CWE-60EF is likely associated with its multi-component phytochemical composition. Xanthine dehydrogenase (XDH), which is interconvertible with xanthine oxidase (XOD), is a key enzyme in purine metabolism and represents an important target in hyperuricemia-related research [22,23]. Recent studies integrating computational and experimental approaches have shown that flavonoid-rich natural products can interact with XDH and contribute to urate-lowering effects [24].

In the present study, molecular docking of 50 identified constituents against five prioritized hub targets, followed by 50 ns molecular dynamics (MD) simulations, highlighted six candidate compounds (mol9, mol25, mol30, mol40, mol41, and mol48) with stable interactions with XDH (Figure 4). During the MD simulations, these ligands remained conformationally stable within the XDH binding region, with RMSD values reaching equilibrium within an acceptable range (Figure 4c,d). Further interaction analysis indicated that these compounds formed hydrogen bonds and hydrophobic interactions with several key residues, including ASP594, ARG32, LYS269, GLU679, and ARG233 (Figure 4b). Although some of these constituents may occupy binding regions distinct from the classical febuxostat-binding site, the simulation results support the structural feasibility of XDH targeting by multiple components of CWE-60EF [25,26]. Importantly, the high binding stability of major constituents such as magnoflorine and phloretin observed in our simulations provides a direct molecular basis for the significant inhibition of XOD activity and reduction of UA levels measured in our zebrafish and HK-2 cell models, thereby linking the predicted chemical-target interactions to our own experimental outcomes.

Several identified constituents also show reported activities consistent with the biological effects observed in this study. Phloretin has been reported to reduce serum uric acid levels through competitive inhibition of URAT1 [27] and to exert anti-inflammatory activity in different experimental settings, including suppression of TLR2/MYD88/NF-kB signaling [28]. These reported effects are in agreement with the observed modulation of urate transport-related genes after CWE-60EF treatment. Magnoflorine, another representative constituent of CWE-60EF, has been shown to alleviate renal tubular injury and chronic nephropathy in multiple models by reducing oxidative stress and inflammatory responses [29,30], and to regulate inflammatory signaling pathways associated with IL-6 and TNF-alpha expression [31]. Taken together, these reports support the view that the phytochemical composition of CWE-60EF may collectively contribute to its anti-hyperuricemic, antioxidant, anti-inflammatory, and renoprotective effects. Our study further substantiates these literature-based claims by demonstrating a consistent synergy between computational docking scores and the observed pharmacological potency of these specific alkaloids and flavonoids in our biological assays.

When viewed in a broader pharmacological context, the anti-hyperuricemic profile of CWE-60EF exhibits distinct and potentially synergistic advantages compared to other traditional gout-treating herbs. For instance, recent studies highlight that the flavonoid-rich fraction from Smilax glabra Roxb. primarily ameliorates uric acid nephropathy by up-regulating renal transporters such as ABCG2, OAT1, OCT2, and OCTN2, thereby facilitating urate excretion [32]. In contrast, Plantago asiatica L. exerts its protective effects via the gut-kidney axis, where it modulates the gut microbiota (notably enriching Lachnospiraceae) to inhibit the activation of the NLRP3 inflammasome [33]. While these herbs focus on specific pathways like renal excretion or microbiota-driven inflammation, the unique chemical matrix of CWE-60EF—characterized by the coexistence of alkaloids (e.g., magnoflorine) and flavonoids (e.g., phloretin)—allows for an integrated multi-target intervention. This suggests that CWE-60EF could provide a more comprehensive therapeutic strategy by simultaneously targeting xanthine oxidase activity, inflammatory signaling, and potentially renal urate handling.

### 3.2. Genetic Support for the Biological Relevance of IL6 in Gout-Related Inflammation

A notable finding of this study is the identification of IL6 as an inflammation-related factor with potential relevance to gout risk, supported by Mendelian randomization (MR) analysis. Although IL-6 is widely recognized as a systemic inflammatory mediator, its relevance to gout has not always been evaluated from a genetic perspective. In our two-sample MR analysis, genetically predicted circulating IL-6 levels were positively associated with gout risk (OR = 1.049), providing supportive evidence for its biological relevance in this disease context.

Gout is an autoinflammatory disorder characterized by monosodium urate (MSU) crystal deposition, which triggers acute inflammatory responses and induces the release of cytokines including IL-6 [34,35]. In this context, our MR findings provide genetic support for the involvement of IL6 in gout-related inflammation and offer a useful framework for interpreting the anti-inflammatory effects observed for CWE-60EF.

In addition, CIBERSORT analysis revealed that IL6 expression was positively associated with resting CD4^+^ memory T cells and activated NK cells, while negative correlations were observed with naive B cells and M0 macrophages. This observation is consistent with the emerging view that immune and metabolic disturbances are closely linked in hyperuricemia-associated tissue injury [36,37]. However, it should be emphasized that MR does not demonstrate that CWE-60EF directly targets IL6. Rather, it supports the biological relevance of the IL6-associated inflammatory context to the disease phenotype, thereby strengthening the interpretation of the observed anti-inflammatory effects of CWE-60EF. While the MR analysis establishes the causal relevance of the IL6-mediated pathway in human gout risk, it does not imply a direct physical interaction between CWE-60EF and the IL6 protein itself. Rather, our results suggest that the pharmacological modulation of IL6 and IL1B by the fraction effectively targets a genetically verified pathway that is central to the disease’s progression.

### 3.3. Modulation of PPAR-Related Regulators and Urate Transport-Related Genes

Hyperuricemia-associated renal tubular injury is accompanied by metabolic disturbance and inflammatory activation. In the present study, UA exposure was associated with reduced expression of PPAR-related regulators, together with increased inflammatory stress, indicating disrupted metabolic homeostasis in HK-2 cells. Treatment with CWE-60EF partially reversed these changes, including restoration of PPARG and PPARA expression. This pattern suggests that CWE-60EF may help improve metabolic balance under hyperuricemic conditions.

PPARs are important regulators of lipid metabolism, oxidative stress, and inflammation, and accumulating evidence also links them to urate transport regulation [38,39]. The observed recovery of PPARG and PPARA expression after CWE-60EF treatment is therefore consistent with a broader protective effect on metabolic and inflammatory disturbances. At the same time, CWE-60EF downregulated the expression of URAT1 and GLUT9, two major urate reabsorption-related transporters, suggesting a coordinated effect on urate transport homeostasis.

Taken together, these findings indicate that the protective effects of CWE-60EF are associated with the combined modulation of PPAR-related metabolic regulators, inflammation-related factors, and urate transport-related genes, rather than with a single defined molecular pathway. This multi-target pattern is consistent with the complex phytochemical nature of the fraction and supports its potential value as a plant-derived bioactive resource for the management of hyperuricemia-associated metabolic disturbances. Nonetheless, at this stage, it remains to be determined whether the restoration of PPARA and PPARG expression represents a primary pharmacological effect via direct ligand-receptor interaction or a secondary physiological recovery following the overall reduction in cellular oxidative stress and inflammatory mobilization. Regardless of the initial trigger, the recovery of PPAR signaling likely provides a crucial feedback mechanism that stabilizes urate transport homeostasis.

### 3.4. In Vivo Relevance and Potential Diagnostic Implications

The biological relevance of CWE-60EF was further supported in the zebrafish model, in which the fraction improved hyperuricemia-associated phenotypes and renal injury-related indicators. In particular, CWE-60EF restored the oat1-related excretion pattern and modulated aberrant hnf4a expression. As HNF4A is an important regulator of hepatic and renal metabolic processes, these findings further support the broad metabolic regulatory effects of CWE-60EF in vivo.

In addition, several representative hub genes, particularly IL6, PPARA, and PTGS2, showed excellent discriminative performance in the current dataset, each achieving an AUC of 1.000. The nomogram constructed from these genes also demonstrated satisfactory calibration. While this result suggests that the identified target set may have potential diagnostic relevance, it should be interpreted with caution. Independent validation in external cohorts will be required before any broader diagnostic application can be considered [40]. The exceptionally high AUC values (1.000) obtained for IL6, PPARA, and PTGS2 warrant critical reflection; these results are likely a consequence of the relatively small clinical discovery cohort and the high degree of disease specificity in the captured samples, which may lead to unintended overfitting. Thus, these signatures are currently more appropriately viewed as prioritized biological candidates associated with hyperuricemic processes rather than definitive clinical biomarkers.

### 3.5. Strengths and Limitations

This study has several strengths. First, it combined phytochemical profiling, transcriptomic and bioinformatics analyses, Mendelian randomization, molecular docking and molecular dynamics simulations, and validation in both cell and zebrafish models, enabling a relatively comprehensive evaluation of CWE-60EF from chemical characterization to biological activity. Second, by integrating chemical data with multi-level biological evidence, the study provided a connected framework for understanding how a plant-derived bioactive fraction may influence hyperuricemia-associated metabolic and inflammatory disturbances. Third, the incorporation of Mendelian randomization added genetic context to the interpretation of inflammation-related targets, particularly IL6.

Several limitations should also be acknowledged. Although the study integrated multiple lines of evidence, the mechanistic conclusions are mainly supported by transcriptional expression changes, computational predictions, and pharmacological observations. While the significant phenotypic improvements observed in both cellular and zebrafish models strongly imply the successful translation of these alterations into functional biological benefits, the current investigation lacks direct downstream validations at the post-translational level. Exploring these targeted functional and protein-level dynamics—such as specific cytokine secretion profiles or localized transporter activities—falls outside our current comprehensive evaluation framework. Therefore, dedicated functional validations represent a vital direction for future independent studies to establish more definitive molecular mechanisms. In addition, the diagnostic model was developed and evaluated within the current dataset, and its generalizability remains to be verified in independent cohorts. Furthermore, while the results support the biological activity of CWE-60EF as a fraction, the contribution of individual constituents and their potential synergistic interactions require further investigation. Finally, while the two selected concentrations produced significant effects across our models, the current study lacks a multi-gradient dose–response relationship in the in vivo zebrafish model, which limits our ability to define the precise therapeutic window for CWE-60EF.

## 4. Materials and Methods

### 4.1. Chemicals and Reagents

The dried roots and rhizomes of *Clematis chinensis* Osbeck were purchased from Hebei Hancao Tang Pharmaceutical Co., Ltd. (Anguo, China). The botanical origin of the plant material was independently authenticated by Professor Hongjiang Jiang at Wendeng Orthopedic Hospital (Weihai, China). A voucher specimen (No. CC-2025-03) was deposited in the Traditional Chinese Medicine Pharmacy of Wendeng Orthopedic Hospital. All reagents used in the study were of analytical grade (Purity > 98%), and their catalog numbers and lot numbers are specified in the Supplementary Materials.

3-(4,5-Dimethylthiazol-2-yl)-2,5-diphenyltetrazolium bromide (MTT, Cat. No. M8180, Lot No. 5550516005, purity ≥ 98%) was purchased from Solarbio (Beijing, China). Allopurinol (Cat. No. S45825, Lot No. JS249998), adenosine (Cat. No. S18049, Lot No. JS312702), potassium oxonate (PO, Cat. No. S17112, Lot No. JS250846), xanthine sodium salt (XSS, Cat. No. T87789, Lot No. KT385574), and xanthine (Cat. No. S18024, Lot No. A23GS158367) were obtained from Yuanye Bio-Technology (Shanghai, China) and Macklin Biochemical Co., Ltd. (Shanghai, China). Uric acid (UA, Cat. No. A011213, Lot No. 9PEAOROE) and HPLC-grade solvents were purchased from Energy Chemical (Shanghai, China).

Reference standards, including magnoflorine (Cat. No. HA061308, Lot No. HR315W20), corosolic acid (Cat. No. HC023143, Lot No. HR2462B1), ursolic acid (Cat. No. HU023144, Lot No. HR425W12), 18β-glycyrrhetinic acid (Cat. No. HG042903, Lot No. HR16307S1), esculin (Cat. No. HE031871, Lot No. HR1176W3), phloretin, and oleanolic acid, were purchased from Shaanxi Tianduoli Biotechnology Co., Ltd. (Xi’an, China). All standards had a purity of ≥98% as determined by HPLC.

The Lactate Dehydrogenase (LDH, Cat. No. A020-2-2, Lot No. 20240925), nitric oxide (NO, Cat. No. A012-1-1, Lot No. 20241012), and creatinine (Cr, Cat. No. ADS-W-FM034, Lot No. ADS20250523) assay kits were purchased from Nanjing Jiancheng Bioengineering Institute (Nanjing, China). The reactive oxygen species (ROS, Cat. No. S0033S, Lot No. A1472503110) assay kit was obtained from Beyotime Biotechnology (Shanghai, China). Details of other analytical grade chemicals and biochemical detection kits are provided in Supplementary Note S1.

### 4.2. Preparation and Chemical Characterization of CWE-60EF

#### 4.2.1. Bio-Guided Fractionation and Preparation

The dried material of *Clematis chinensis* Osbeck (100 g) was extracted with 1 L of deionized water under reflux for 1 h. The extract was filtered and concentrated under reduced pressure to obtain the crude extract.

The crude extract was subjected to D101 macroporous resin column chromatography and sequentially eluted with an ethanol–water gradient (0%, 20%, 40%, 60%, and 80%, *v*/*v*).

To prepare the samples for biological evaluation, the CWE-60EF fraction was concentrated under reduced pressure and lyophilized to obtain a stable powder, which was stored in a desiccator. Prior to cell treatment, the powder was dissolved in ultrapure water to prepare a concentrated stock solution. The pH of the solution was adjusted to 7.2–7.4 to ensure compatibility with the cellular environment, followed by sterilization through a 0.22 μm membrane filter. To avoid potential artifacts caused by alterations in pH, osmolarity, or nutrient concentration, the final concentration of the aqueous stock in the treatment medium was maintained at a very low ratio (less than 1%, *v*/*v*) for all subsequent assays.

#### 4.2.2. Qualitative Analysis of CWE-60EF via HR-LC-MS/MS

Chemical characterization of CWE-60EF was performed using a high-resolution Q Exactive Orbitrap mass spectrometer (Thermo Fisher Scientific, Waltham, MA, USA). Chromatographic separation was achieved on an ACQUITY UHPLC BEH C18 column (2.1 × 75 mm, 1.7 μm). To ensure chemical standardization and reproducibility, seven major markers were quantified using a triple-quadrupole LC-MS/MS system (Shimadzu LCMS-8050; Shimadzu, Kyoto, Japan) as confirmed with authentic reference standards. Detailed instrumental settings, including ionization parameters, fragmentation pathways, and quantitative validation methods, are documented in Supplementary Note S4. Identification was based on accurate mass measurements (±5 ppm), fragmentation patterns, and database matching.

Quantitative analysis was conducted on a Shimadzu LCMS-8050 system using Multiple Reaction Monitoring (MRM). Technical parameters, including regression equations and MRM transitions for the quantified markers, are provided in Supplementary Table S4.

### 4.3. Integrative Bioinformatics and Network Pharmacology

#### 4.3.1. Weighted Gene Co-Expression Network Analysis (WGCNA) and Hub Gene Identification

The gout-related gene expression dataset GSE160170 was retrieved from the Gene Expression Omnibus (GEO) database and included 12 samples, comprising 6 gout cases and 6 control samples. Differentially expressed genes (DEGs) were identified using the limma package (version 3.58.1) in R [41], using a threshold of adjusted *p* < 0.05 and log_2_Fold Change > 1 to ensure statistical and biological relevance.

Weighted gene co-expression network analysis (WGCNA) was performed using the WGCNA package (version 1.72) in R to identify gene modules associated with the disease phenotype [42]. A soft-thresholding power of 12 was selected to construct a scale-free co-expression network, resulting in a scale-free topology fit index (R^2^) greater than 0.85. Among the generated modules, MEdarkturquoise was identified as the key module for subsequent analysis.

Predicted targets of the identified constituents in CWE-60EF were obtained from SwissTargetPrediction and SEA with the species restricted to Homo sapiens [43,44]. Gout-related targets were collected from disease databases, including GeneCards, DisGeNET, and OMIM, using “gout” and “hyperuricemia” as search keywords. After merging the results and removing duplicate entries, candidate genes were identified as the intersection of four datasets: DEGs from GSE160170, genes in the MEdarkturquoise module identified by WGCNA, predicted targets of CWE-60EF constituents, and gout-related targets from disease databases. These overlapping genes were used for subsequent network and enrichment analyses.

To further explore the interactions among these candidate genes, a protein–protein interaction (PPI) network was constructed based on the STRING database using a confidence score threshold of 0.7. The resulting network was imported into Cytoscape (version 3.9.1) for visualization and analysis [45]. Hub genes were prioritized using the CytoHubba plugin in Cytoscape based on topological analysis [46]. IL6 showed the highest network connectivity and was regarded as the key hub gene. In addition, five representative hub genes (IL6, PPARA, PPARG, XDH, and PTGS2) were selected based on network topology and biological relevance for downstream expression validation, ROC analysis, and diagnostic model construction.

#### 4.3.2. Nomogram Construction and Immune Infiltration Analysis

Based on the five representative hub genes (IL6, PPARA, PPARG, XDH, and PTGS2) identified above, a diagnostic nomogram model was constructed to evaluate their potential diagnostic value in gout. For nomogram construction, the expression value of each selected gene was dichotomized into high- and low-expression groups according to the median expression level across all samples. A multivariable logistic regression model was then established using the rms package (version 7.0-0) in R, and a nomogram was generated to quantitatively estimate gout risk. Model calibration was assessed by bootstrap resampling.

In addition, immune infiltration analysis was performed using the CIBERSORT algorithm (version 1.03) to estimate the relative proportions of 22 immune cell types in gout and control samples [47]. The normalized gene expression matrix from GSE160170 was used as input, and the LM22 signature matrix was applied with 1000 permutations. Since IL6 was identified as the key hub gene with the highest network connectivity, correlations between IL6 expression and immune cell proportions were evaluated using Spearman’s rank correlation analysis.

#### 4.3.3. Molecular Docking and Molecular Dynamics Simulation

Molecular docking was conducted using the Molecular Operating Environment (MOE) 2019.0102 platform to investigate interactions between 50 identified constituents and five hub targets (IL6, PPARA, PPARG, XDH, and PTGS2). Receptor structures were retrieved from the RCSB Protein Data Bank (PDB IDs: 4J4L, 6KAX, 9F7W, 2CKJ, and 5F19). To assess the dynamic stability of the prioritized ligand–protein complexes, 50 ns molecular dynamics (MD) simulations were performed using GROMACS 2023.2 with the CHARMM36 force field. Trajectory analyses, including RMSD, RMSF, and hydrogen bond persistence, were utilized to evaluate structural equilibrium. All technical procedures and specific parameter configurations for docking and MD simulations are detailed in Supplementary Note S5.

### 4.4. Two-Sample Mendelian Randomization Analysis

To investigate the potential causal effect of circulating interleukin-6 (IL-6) levels on gout risk, a two-sample Mendelian randomization (2SMR) analysis was conducted in R using the TwoSampleMR (version 0.6.1) and vcfR packages (version 1.16.0) [48,49]. Instrumental variables (IVs) were selected in accordance with the three core assumptions of Mendelian randomization [50]: robust association with the exposure, independence from potential confounders, and absence of any direct effect on the outcome except through the exposure. Genetic instruments for circulating IL-6 were obtained from the genome-wide association study (GWAS) summary statistics of circulating proteins (ID: prot-b-2) from a publicly available dataset [51], whereas gout outcome data were retrieved from the FinnGen consortium (ID: finn-b-M13_GOUT) [52]. Single nucleotide polymorphisms (SNPs) associated with circulating IL-6 at *p* < 5 × 10^−6^ were initially selected. This threshold was chosen to ensure a sufficient number of instrumental variables (IVs) for a robust MR analysis, as protein-exposure GWAS often yield limited SNPs at the stringent *p* < 5 × 10^−8^ level. To ensure instrument independence and minimize linkage disequilibrium (LD), SNPs were clumped using a 10,000 kb window and an *r*^2^ threshold of <0.001 based on the 1000 Genomes Phase 3 European reference panel. After clumping, 15 independent SNPs were retained as IVs (Table S1). The exposure and outcome datasets were then harmonized using the harmonise_data function to align effect alleles. The primary causal estimate was calculated using the inverse variance weighted (IVW) method. Sensitivity analyses, including MR-Egger regression, weighted median estimation, and leave-one-out analysis, were further performed to evaluate the robustness of the results. Heterogeneity among SNPs was assessed using Cochran’s Q test, and horizontal pleiotropy was examined using the MR-Egger intercept test. Results were reported as odds ratios (ORs) with 95% confidence intervals (CIs). Instrument strength was evaluated using the F-statistic (*F* = [*β*/*SE*]^2^), and the mean F value was 28.53, indicating a low risk of weak instrument bias.

### 4.5. Adenosine-Induced Metabolic Dysfunction in HK-2 Cells

#### 4.5.1. Cell Model Establishment and Drug Treatment

HK-2 cells were cultured in RPMI 1640 medium (Gibco, Grand Island, NY, USA) supplemented with 10% fetal bovine serum (FBS) and 1% penicillin-streptomycin. The cells were maintained in a humidified incubator at 37 °C with 5% CO_2_. The culture medium was replaced every 48 h, and cells were passaged using 0.25% trypsin-EDTA upon reaching 80–90% confluence.

To mimic enhanced purine catabolism and uric acid (UA) production, an adenosine-induced metabolic dysfunction model was established as previously described with minor modifications [53]. Cells were seeded into 96-well or 6-well plates and allowed to attach for 24 h before treatment. For drug intervention, cells were pretreated with CWE-60EF (80 or 320 µg/mL) or allopurinol (100 µM, as a positive control) for 24 h. Subsequently, adenosine (10 mmol/L) was added and incubated for an additional 24 h to promote the intracellular accumulation of purine metabolites. Thereafter, xanthine oxidase (XOD, 0.005 U/mL) was introduced to catalyze the conversion of purine intermediates into UA. Control cells received an equivalent volume of vehicle only.

#### 4.5.2. Measurement of Uric Acid Production and HGPRT Activity

Following treatment, culture supernatants were collected for determination of UA levels using high-performance liquid chromatography (HPLC), as described in Supplementary Note S3. In parallel, cell pellets were harvested for the assessment of hypoxanthine–guanine phosphoribosyltransferase (HGPRT) activity and content. HGPRT, a key enzyme in the purine salvage pathway, was measured to evaluate the potential regulatory effect of CWE-60EF on purine reutilization.

#### 4.5.3. Assessment of Oxidative Stress

Intracellular reactive oxygen species (ROS) levels were determined using the DCFH-DA fluorescent probe according to the manufacturer’s instructions. To further evaluate the cellular antioxidant status, the activities of superoxide dismutase (SOD) and catalase (CAT) were measured using commercial assay kits. These assays were performed to assess oxidative stress and antioxidant defense capacity in HK-2 cells following adenosine induction and drug treatment.

### 4.6. UA-Induced Cell Injury Model and Mechanistic Validation

To mimic hyperuricemia-associated cellular stress and to validate the transport-, metabolism-, and inflammation-related markers identified by integrative analyses, a uric acid (UA)-induced cell injury model was established [54].

#### 4.6.1. Model Establishment and Functional Assessment

Based on previous reports and our preliminary cytotoxicity screening, 800 μg/mL UA was selected to establish the cell injury model. To prepare the UA solution, UA powder was dissolved in 0.1 M NaOH at 60 °C according to established protocols [55]. To avoid alkaline-induced cytotoxicity, the solution was carefully neutralized to a physiological pH of 7.4 using HCl and sterile-filtered before application. Microscopic observation confirmed the absence of urate crystal formation during the incubation period. After treatment, cell viability was determined using the MTT assay according to the manufacturer’s instructions. Lactate dehydrogenase (LDH) release and nitric oxide (NO) levels in the culture supernatant were further measured to evaluate membrane damage and oxidative/inflammatory stress, respectively.

#### 4.6.2. Transcriptional Validation of the Transporter–Regulatory Axis

To investigate the regulatory effects of CWE-60EF on key genes, RT-qPCR was performed to assess the transcriptional expression of a six-gene panel selected from previous integrative analyses. These genes were classified into three functional categories: (1) urate transporters, including URAT1 (SLC22A12) and GLUT9 (SLC2A9); (2) metabolic regulators, including PPARA and PPARG; and (3) inflammatory mediators, including IL-6 and IL1B. Total RNA extraction and cDNA synthesis were performed as described above. Primer sequences synthesized by Sangon Biotech (Shanghai, China) are listed in Supplementary Table S5.

#### 4.6.3. Intracellular ROS Detection

Intracellular reactive oxygen species (ROS) levels were measured using the DCFH-DA fluorescent probe. Briefly, after the indicated treatments, cells were incubated with 10 μM DCFH-DA at 37 °C for 30 min in the dark, followed by washing with PBS to remove excess probe. Fluorescence signals were observed under a fluorescence microscope, and representative images were captured. Where applicable, fluorescence intensity was further quantified to evaluate intracellular ROS accumulation.

## 5. Conclusions

This study provides a comprehensive pharmacological evaluation of CWE-60EF, a bioactive fraction from *Clematis chinensis* Osbeck, demonstrating its potent anti-hyperuricemic, renoprotective, and anti-inflammatory properties across zebrafish and HK-2 cell models. A key contribution of this work is the novel integration of phytochemical profiling, multi-level bioinformatics, and human genetic evidence (Mendelian randomization), which collectively bridges the gap between traditional ethnopharmacology and evidence-based genomic medicine.

Our findings reveal that CWE-60EF restores metabolic and inflammatory homeostasis by modulating a complex network involving XDH inhibition, PPAR signaling, and the IL-6-mediated inflammatory axis. Future research should focus on direct target validation using genetic perturbation models (e.g., CRISPR/Cas9 or RNAi) and evaluate the clinical translational potential of this standardized fraction in larger patient cohorts. Ultimately, this study reinforces the value of *C. chinensis* as a promising plant-derived therapeutic resource for managing the metabolic disturbances associated with hyperuricemia and gout.

## Figures and Tables

**Table 1:** Quantitative contents (μg/mL) of 7 marker compounds in three independent batches of CWE-60EF (*n* = 3).

Compound	Batch 1	Batch 2	Batch 3	Mean Content ± SD (μg/mL)
Magnoflorine	20.15	35.75	48.9	34.93 ± 14.39
Phloretin	12.96	14.9	15.67	14.51 ± 1.40
Corosolic acid	0.75	0.98	0.88	0.87 ± 0.11
Ursolic acid	0.58	0.68	0.63	0.63 ± 0.05
Oleanolic acid	0.57	0.56	0.61	0.58 ± 0.03
18β-Glycyrrhetinic acid	0.24	0.21	0.22	0.22 ± 0.02
Esculetin	N.D.	N.D.	N.D.	N.D.

Notes: N.D. = Not Detected.

## Data Availability

The data presented in this study are available in the article and Supplementary Materials. Additional data are available from the corresponding author upon reasonable request.

## Supplementary Materials

The following supporting information can be downloaded at: https://www.mdpi.com/article/10.3390/ph19060830/s1, Supplementary Note S1: Complete list of reagents and consumables; Supplementary Note S2: Formula and parameters for the calculation of cell viability; Supplementary Note S3: Quantitative method and validation for uric acid determination; Supplementary Figure S1: Calibration curve and linearity of uric acid standard; Supplementary Note S4: Instrumental parameters for LC-HRMS/MS analysis; Supplementary Figure S2: Proposed MS/MS fragmentation pathways of representative compounds; Supplementary Figure S3: Representative LC-MS/MS chromatograms (TIC) of three independent batches of CWE-60EF; Supplementary Table S1: Detailed information of instrumental variables (IVs) for IL-6 and gout; Supplementary Table S2: Summary of MR estimates, heterogeneity, and pleiotropy analysis; Supplementary Table S3: Characterization of chemical constituents in the CWE-60EF fraction via HRMS; Supplementary Table S4: MRM transitions and calibration curves for quantitative detection; Supplementary Note S5: Detailed protocols for molecular docking and molecular dynamics simulations; Supplementary Table S5: Sequences of primers used for RT-qPCR analysis; Supplementary Figure S4. Cross-validation of CWE-60EF cytotoxicity using the CCK-8 assay.

## Abbreviations

The following abbreviations are used in this manuscript:

2SMR	two-sample Mendelian randomization
ANOVA	analysis of variance
AUC	area under the curve
CAT	catalase
CI	confidence interval
CKD	chronic kidney disease
Cr	creatinine
CWE	Clematis chinensis water extract
CWE-60EF	60% ethanol fraction of Clematis chinensis
DEGs	differentially expressed genes
GLUT9	glucose transporter 9
HGPRT	hypoxanthine-guanine phosphoribosyltransferase
HPLC	high-performance liquid chromatography
HUA	hyperuricemia
IL-6	interleukin-6
IVW	inverse variance weighted
LDH	lactate dehydrogenase
MD	molecular dynamics
MTT	3-(4,5-dimethylthiazol-2-yl)-2,5-diphenyltetrazolium bromide assay
NO	nitric oxide
OR	odds ratio
PBS	phosphate-buffered saline
PPAR	peroxisome proliferator-activated receptor
PPARA	peroxisome proliferator-activated receptor alpha
PPARG	peroxisome proliferator-activated receptor gamma
PTGS2	prostaglandin-endoperoxide synthase 2
ROC	receiver operating characteristic
ROS	reactive oxygen species
RT-qPCR	real-time quantitative polymerase chain reaction
SNP	single nucleotide polymorphism
SOD	superoxide dismutase
UA	uric acid
ULT	urate-lowering therapy
URAT1	urate transporter 1
WGCNA	weighted gene co-expression network analysis
XDH	xanthine dehydrogenase
XOD	xanthine oxidase
