# Supplementary material for: Clematis Chinensis Attenuates Hyperuricemia Through the Coordinated Regulation of Purine Metabolism and Inflammatory Responses: An Integrative Study
Source: Pharmaceuticals (Basel). 2026 May 26;19(6):830. doi: 10.3390/ph19060830 (PMC13304788; doi:10.3390/ph19060830)
Supplement: Supplementary file 1 [file pharmaceuticals-19-00830-s001.zip › Supplementary_Information.pdf]

Supplementary Information

Manuscript Title: Clematis chinensis Attenuates Hyperuricemia through Coordinated Regulation of Purine Metabolism and Inflammatory Responses: An Integrative Study

Ze Fu, Hao Ju, Zi-Hao Chen, Yan-Chao Wu, Hui-Jing Li *

*Corresponding author: lihj@hit.edu.cn

Supplementary Note S1. Complete List of Reagents and Consumables

Fetal bovine serum (FBS; Cat. No. 164210-50, Lot No. SA241025) was obtained from Procell (Wuhan, China). RPMI 1640 medium (Cat. No. 31800, Lot No. 2500120022) was supplied by Solarbio (Beijing, China), and phosphate-buffered saline (PBS) was purchased from Servicebio (Wuhan, China).

The BCA Protein Assay Kit (Cat. No. PC0020, Lot No. 240008023), Superoxide Dismutase (SOD) Assay Kit (Cat. No. BC0175, Lot No. 2408006), and Catalase (CAT) Assay Kit (Cat. No. BC4785, Lot No. 2407005) were purchased from Solarbio (Beijing, China).

Hypoxanthine-guanine phosphoribosyltransferase (HGPRT) Assay Kit was sourced from Meike Biotech (Shanghai, China). Tricaine solution, Sodium hydroxide (NaOH) solution, and Phosphoric acid were purchased from GD Tech (Taipei, Taiwan). HPLC-grade methanol, acetonitrile, and formic acid were obtained from Energy Chemical (Shanghai, China). Macroporous adsorption resin D101 was provided by Solarbio (Beijing, China). RNA-simple Total RNA Extraction Kit and FastKing One-Step RT-qPCR Kit were sourced from Tiangen Biotech (Beijing, China). Chromatographic analysis was performed using an Agilent ZORBAX SB-C18 HPLC column (4.6 mm × 250 mm, 5 μm).

Supplementary Table S1. Detailed Information of Instrumental Variables (IVs) for IL-6 and Gout

SNP	Effect Allele (EA)	Other Allele (OA)	Beta (Exposure)	Beta (Outcome)	EAF	SE (Outcome)	P-value (Outcome)	Chromosome	Position (bp)	SE (Exposure)	P-value (Exposure)	F-statistic
rs112840232	C	A	0.5233	0.1265	0.0157	0.1095	reported	9	130615272	0.0974	8.21996559903803e-08	28.86
rs137923165	A	G	1.3187	0.2758	0.0051	0.1416	reported	2	49242664	0.2661	7.57791971868786e-07	24.55
rs138611179	A	G	2.3599	-0.5986	0.0013	0.7313	reported	2	21036377	0.4754	7.23602402280251e-07	24.64
rs138672581	G	C	1.2387	0.0709	0.0036	0.2098	reported	3	64926905	0.2415	3.06902198839116e-07	26.31
rs140359048	T	C	1.7671	-0.0882	0.0024	0.1136	reported	1	72620933	0.3461	3.47096312647506e-07	26.06
rs145871805	G	A	1.4046	0.2754	0.0054	0.1065	reported	5	132653232	0.235	2.5299969638711e-09	35.72

rs17631606	T	C	1.8896	0.0869	0.0035	0.1599	reported	13	59859887	0.3578	1.36000341846013e-07	27.89
rs181789617	G	A	2.4808	0.1293	0.0016	0.1645	reported	10	96257957	0.4966	6.14893638512627e-07	24.95
rs182643426	A	G	2.0695	-0.0158	0.0028	0.097	reported	5	61532029	0.3412	1.45999039952902e-09	36.78
rs189753081	T	C	0.8933	-0.0404	0.0067	0.3698	reported	2	65559954	0.1755	3.77198537333025e-07	25.91
rs190592371	G	A	2.4124	0.1296	0.0017	0.1646	reported	10	96193514	0.485	6.87796560987083e-07	24.74
rs191222133	A	G	2.7955	-0.245	6e-04	0.5389	reported	6	20653991	0.4989	2.27101498570377e-08	31.39
rs192792066	T	A	3.3603	0.4952	8e-04	0.8452	reported	11	8383553	0.6715	5.89603428162787e-07	25.04
rs4845373	T	C	0.1349	0.0104	0.3636	0.0274	reported	1	154417829	0.0254	1.15800384123579e-07	28.21

rs72627143	A	G	4.5582	0.3985	7e-04	0.3437	reported	14	74431428	0.7507	1.40601514906196e-09	36.86

Supplementary Table S2. Summary of MR Estimates, Heterogeneity, and Pleiotropy Analysis

Method	NSNP	Beta (SE)	OR (95% CI)	P-value	Heterogeneity (Q P)	Pleiotropy (Intercept P)
Inverse variance weighted	15	0.0476 (0.023)	1.049 (1.002, 1.098)	0.040	0.558	–
MR Egger	15	0.0391 (0.029)	1.040 (0.983, 1.100)	0.198	0.498	0.632
Weighted median	15	0.0491 (0.032)	1.050 (0.986, 1.119)	0.128	–	–
Simple mode	15	0.0511 (0.049)	1.052 (0.956, 1.159)	0.317	–	–
Weighted mode	15	0.0265 (0.034)	1.027 (0.961, 1.098)	0.450	–	–

Supplementary Note S2. Formula and Parameters for the Calculation of Cell Viability

$$\text{Cell viability（\%）} = \frac{\text{A\_drug- A\_blank control}}{\text{A\_normal control - A\_blank control}} \times 100\%$$

Among them, A_drug represents the absorbance of cells after drug treatment; A_normal control group denotes the solvent control group; and A_blank control group refers to the absorbance measured in wells containing only culture medium and MTT reagent without cells (used to subtract the background absorbance).

Supplementary Note S3. Quantitative Method and Validation for Uric Acid Determination

An accurately weighed amount of uric acid (12.64 mg) was transferred into a 25 mL volumetric flask. The sample was dissolved in 0.1 M NaOH solution and diluted to the mark to prepare a stock standard solution. This stock solution was further diluted with ultrapure water to establish a series of working standard solutions at concentrations of 3.95, 7.9, 15.8, 31.6, 63.2, 126.4, 252.8, and 505.60 $\mu g \cdot mL^{-1}$. For each concentration level, 20 μL aliquots were precisely injected into the chromatographic system under identical analytical conditions. The corresponding peak areas were determined and

recorded. A linear regression analysis was performed using uric acid concentration (μg·mL⁻¹) as the independent variable (x-axis) and peak area as the dependent variable (y-axis) to construct the calibration curve (see Figure S1).

Chromatographic conditions (applied to all injections): Instrument: Shimadzu LC-20A (Shimadzu); detection wavelength: 283 nm; column: C18 column (4.6 mm × 250 mm, 5 μm); mobile phase: acetonitrile–0.1% phosphoric acid aqueous solution (3:97, v/v); column temperature: 30 °C; flow rate: 1.0 mL·min⁻¹; injection volume: 20 μL.

Supplementary Figure S1. Calibration Curve and Linearity of Uric Acid Standard

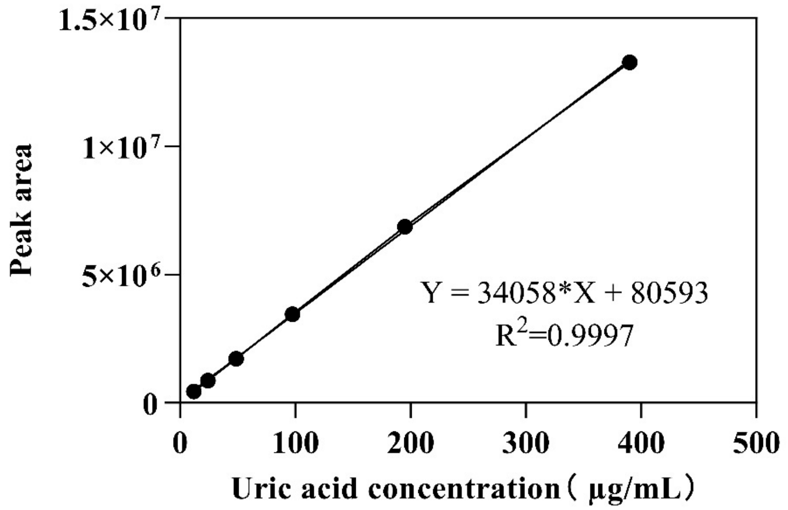

Supplementary Note S4. Instrumental Parameters for LC-HRMS/MS Analysis

1. Qualitative Analysis (HR-LC-MS/MS)

High-resolution mass spectrometry (HRMS) analysis was performed using a Thermo Fisher Scientific Q Exactive Orbitrap MS coupled with a Dionex Ultimate 3000 RSLC system.

Chromatographic Conditions: Analytical column: ACQUITY UHPLC BEH C18 (2.1 × 75mm,1.7 μm); Column temperature: 35°C; Flow rate: 0.3mL/min; Injection volume: 3 μL.

Mobile Phase: A: 0.1% formic acid in water; B: acetonitrile.

Gradient Program: 0 – 30min, 5%→50%B; 30 – 40min, 50%→95%B; 40 – 50min, 95%B.

Mass Spectrometry Settings: ESI positive/negative mode; Scan range m/z 50 – 1500; MS resolution 140,000; MS/MS resolution 17,500.

Source Parameters: Sheath gas: 40; Aux gas: 15; Spray voltage: 3.2kV (+)/2.8kV (-); Capillary temp: 320°C; S-lens RF level: 55.

2. Quantitative Standardization and Reproducibility (Triple-Quadrupole LC-MS/MS)

To ensure batch-to-batch reproducibility as requested by the reviewers, seven marker compounds (Magnoflorine, Phloretin, Corosolic acid, Oleanolic acid, Ursolic acid, Glycyrrhetinic acid, and Esculetin) were quantified.

Instrumentation: Shimadzu LCMS-8050 (Triple Quadrupole) in Multiple Reaction Monitoring (MRM) mode.

Method Validation: A five-point calibration curve (R^2>0.99) was established for each marker. Detailed validation parameters, including MRM transitions, linear regression equations, and limits of detection (LOD/LOQ), are summarized in Supplementary Table S4.

Reproducibility Data: The quantitative results (Mean ± SD) for three independent batches are presented in Table 1 in the main text. Representative LC-MS/MS chromatograms demonstrating batch-to-batch consistency are provided in Supplementary Figure S3.

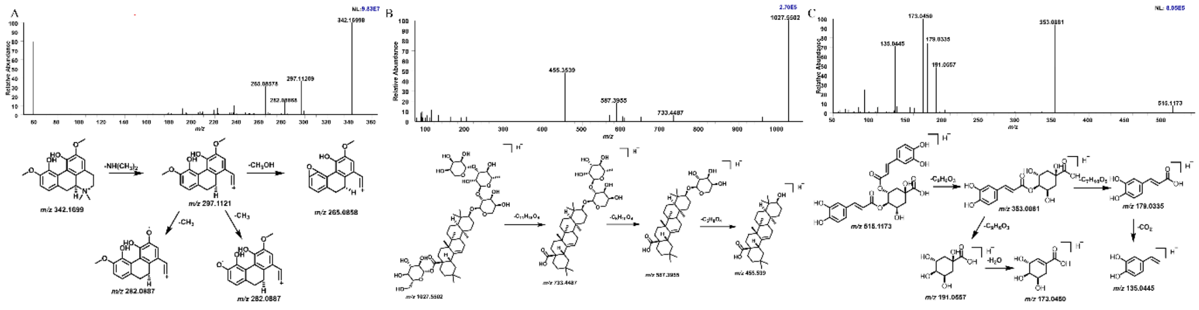

Supplementary Figure S2. Proposed MS/MS Fragmentation Pathways of Representative Compounds

The MS/MS mass spectra and corresponding fragmentation mechanisms of three representative key compounds from the 60% ethanol eluate fraction of Clematis chinensis, including magnoflorine (A), clematichinenoside A (B), and isochlorogenic acid C (C). For each sub-figure, the upper panel presents the MS/MS spectrum of the corresponding compound, displaying the m/z values and relative abundances of major precursor ions and fragment ions.

The lower panel shows the molecular structure of the compound along with its proposed fragmentation pathways, where arrows indicate the cleavage sites of major fragmentation bonds, accompanied by annotations of theoretical m/z values for the corresponding fragment ions.

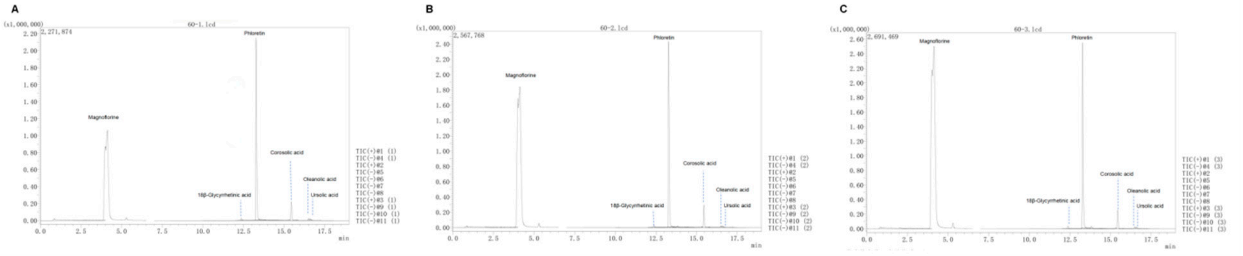

Supplementary Figure S3. Representative LC-MS/MS chromatograms (TIC) of three independent batches of CWE-60EF. (A) Batch 60-1; (B) Batch 60-2; (C) Batch 60-3. The identified marker compounds are labeled at their respective retention times. The high degree of consistency across the three batches demonstrates the chemical standardization and reproducibility of the CWE-60EF preparation.

Supplementary Table S3. Characterization of Chemical Constituents in the CWE-60EF Fraction via HRMS

Peak	Name	Formula	Error (ppm)	Calc. MW	*m/z*	t_R (min)	Reference Ion	MS/MS	Area
1	Malic acid	$C_4H_6O_5$	-3.19	134.0211	133.0138	1.35	$[M-H]^-$	133, 115, 71	440111057
2	Nicotinamide	$C_6H_6N_2O$	2.94	122.0484	123.0557	1.36	$[M+H]^+$	123, 80	155781251
3	Esculin	$C_{15}H_{16}O_9$	1.86	340.0801	339.0729	4.42	$[M-H]^-$	339, 177	77890401
4	Salicylic acid	$C_7H_6O_3$	-4.33	138.0311	137.0238	4.94	$[M-H]^-$	137, 125	2034142687
5	Scoparone	$C_{11}H_{10}O_4$	1.78	206.0583	207.0656	5.35	$[M+H]^+$	207, 161, 133	43385155
6	Gentisic acid	$C_7H_6O_4$	-3.06	154.0261	153.0189	5.43	$[M-H]^-$	153, 109	79705939

7	3,4-Dihydroxyphenylacetic acid	$C_8H_8O_4$	-2.41	168.0419	167.0346	5.64	$[M-H]^-$	167, 152, 123	60817453
8	Isophthalic acid	$C_8H_6O_4$	-2.38	166.0262	165.0189	6.28	$[M-H]^-$	165, 121	220335091
9	Manshuricaterside A or isomer	$C_{21}H_{36}O_{11}$	3.13	464.2252	463.2188	6.35	$[M-H]^-$	463, 331, 191, 175, 113, 101, 71	59444550
10	Fraxetin	$C_{10}H_8O_5$	0.22	208.0372	209.0445	6.49	$[M+H]^+$	209, 194, 149	169717636
11	4-Methoxybenzaldehyde	$C_8H_8O_2$	-0.32	136.0524	137.0597	6.82	$[M+H]^+$	137, 122, 94	82172096

12	4-Methylumbelliferyl-α-D-glucopyranoside	$C_{16}H_{18}O_8$	-0.16	338.1001	339.1074	6.99	$[M+H]^+$	327, 177, 153	345554943
13	Eugenol	$C_{10}H_{12}O_2$	-0.2	164.0837	165.091	7.27	$[M+H]^+$	165, 137, 133, 105	125683366
14	Manshuricaterside A or isomer	$C_{21}H_{36}O_{11}$	2.98	464.2252	463.2189	7.43	$[M-H]^-$	463, 331, 191, 175, 113, 101, 71	299343588
15	Magnoflorine	$C_{20}H_{23}NO_4$	-0.61	341.1625	342.1698	7.45	$[M+H]^+$	342, 297, 282, 265	15972479823
16	Homovanillic acid	$C_9H_{10}O_4$	-1.14	182.057	181.05	7.8	$[M-H]^-$	181, 163,	217194

				7	04	2		137	146
17	Fraxetin	$C_{10}H_8O_5$	0.51	208.0373	209.0446	7.82	$[M+H]^+$	209, 194, 149, 131	6605308
18	Camphor	$C_{10}H_{16}O$	0.03	152.1201	153.1274	8.06	$[M+H]^+$	153, 135, 109, 107	8998360
19	Manshuricaterside A or isomer	$C_{21}H_{36}O_{11}$	2.62	464.2252	463.2186	8.11	$[M-H]^-$	463, 331, 191, 175, 113, 101, 71	148381097
20	Isoferulic acid	$C_{10}H_{10}O_4$	0.28	194.058	195.0653	8.18	$[M+H]^+$	195, 175, 163	432755216

21	Vanillin	$C_8H_8O_3$	1.44	152.0476	153.0548	8.30	$[M+H]^+$	153, 125, 111	100956189
22	Syringic acid	$C_9H_{10}O_5$	-0.69	198.0527	197.0454	8.33	$[M-H]^-$	197, 182, 153, 123, 121	46950933
23	5-methyl-4-{[(2S,3R,4S,5S,6R)-3,4,5-trihydroxy-6-(hydroxymethyl)oxan-2-yl]oxy}-2H-chromen-2-one	$C_{16}H_{18}O_8$	-0.39	338.1000	339.1073	8.57	$[M+H]^+$	339, 177	198114236
24	Carvone	$C_{10}H_{14}O$	0.6	150.1046	151.1118	8.73	$[M+H]^+$	151, 133, 123, 109, 107	107299145
25	Caffeic acid	$C_9H_8O_4$	-0.81	180.0421	179.0348	8.91	$[M-H]^-$	179, 135, 109,107	28808296

26	Manshuricaterside A or isomer	$C_{21}H_{36}O_{11}$	1.95	464.2252	463.2183	9.53	$[M-H]^-$	463, 331, 191, 175, 113, 101, 71	26773388
27	Suberic acid	$C_8H_{14}O_4$	-1.45	174.0890	173.0817	9.64	$[M-H]^-$	173, 111	181620981
28	Jasmone	$C_{11}H_{16}O$	0.4	164.1202	165.1275	9.73	$[M+H]^+$	165, 147, 127, 123	65811375
29	Lariciresinol 4-O-glucoside	$C_{26}H_{34}O_{11}$	1.46	522.2109	567.2094	9.80	$[M+FA-H]^-$	521, 359, 329, 344	442793906
30	Lariciresinol 4-O-glucoside	$C_{26}H_{34}O_{11}$	2.66	522.2115	521.204	10.36	$[M-H]^-$	521, 359, 329,	207756052

								344	
31	3,4,5-trihydroxy-6-(hydroxymethyl)oxan-2-yl 2-(prop-1-en-2-yl)-2,3-dihydro-1-benzofuran-5-carboxylate	$C_{18}H_{22}O_8$	0.3	366.1316	367.1389	10.40	$[M+H]^+$	367, 205	75095682
32	Brosimacutin H	$C_{20}H_{24}O_6$	-0.02	360.1573	361.1646	10.69	$[M+H]^+$	361, 331, 313, 287	112530770
33	Ethyl 4-methoxycinnamate	$C_{12}H_{14}O_3$	0.26	206.0944	207.1016	10.7	$[M+H]^+$	307, 189, 161	114894026
34	Manshuricaterside A or isomer	$C_{21}H_{36}O_{11}$	3.13	464.2252	463.2188	11.01	$[M-H]^-$	463, 331, 191, 175, 113, 101, 71	39458151
35	Ferulic acid	$C_{10}H_{10}O_4$	-1.38	194.0576	193.0503	11.46	$[M-H]^-$	193, 178,	120761352

								134	
36	Manshuricaterside A or isomer	$C_{21}H_{36}O_{11}$	3.01	464.2252	463.2188	11.64	$[M-H]^-$	463, 331, 191, 175, 113, 101, 71	66298118
37	3,5-Dicaffeoylquinic acid	$C_{25}H_{24}O_{12}$	1.99	516.1278	515.1204	11.86	$[M-H]^-$	515, 353 191, 179, 173, 135	38539224
38	Catechin	$C_{15}H_{14}O_{6}$	1.61	290.0795	289.0723	12.20	$[M-H]^-$	289, 259, 215, 173	49957588
39	Manshuricaterside A or isomer	$C_{21}H_{36}O_{11}$	2.62	464.2252	463.2186	12.33	$[M-H]^-$	463, 331,	162617960

								191, 175, 113, 101, 71	
40	3,4-Dimethoxycinnamic acid	$C_{11}H_{12}O_4$	0.1	208.0736	209.0809	13.11	$[M+H]^+$	209, 191, 163	26830505
41	4,5-Dicaffeoylquinic acid	$C_{25}H_{24}O_{12}$	2.27	516.128	515.1207	13.17	$[M-H]^-$	515, 353 191, 179, 173, 135	76031366
42	Phloretin	$C_{15}H_{14}O_5$	-0.07	274.0841	275.0914	13.95	$[M+H]^+$	275, 107	216792154
43	Cimicifugic acid B	$C_{21}H_{20}O_{11}$	1.19	448.1011	447.0939	14.08	$[M-H]^-$	447, 253, 191, 161, 123,	451868660

								109	
44	4-Methylumbelliferone hydrate	$C_{10}H_8O_3$	-0.23	176.0473	177.0546	14.09	$[M+H]^+$	177, 163, 149, 135, 117, 89	295480372
45	Nootkatone	$C_{15}H_{22}O$	0.79	218.1672	219.1745	14.33	$[M+H]^+$	219, 204, 189	59184965
46	Corosolic acid or isomer	$C_{30}H_{44}O_2$	-0.73	436.3336	437.3411	14.92	$[M+H]^+$	439, 391, 247, 201, 191, 189, 159, 119	21139008
47	Oleanonic acid or isomer	$C_{30}H_{46}O_3$	-0.84	454.3441	455.3516	14.92	$[M+H]^+$	455, 437,	127510905

								409, 391, 247, 201, 191, 119	
48	Corosolic acid or isomer	$C_{30}H_{44}O_2$	-1	436.3336	437.341	15.21	$[M+H]^+$	439, 391, 247, 201, 191, 189, 159, 119	17664889
49	Oleanonic acid or isomer	$C_{30}H_{46}O_3$	-0.42	454.3441	455.3518	15.21	$[M+H]^+$	455, 437, 409, 391, 247, 201, 191, 119	50397429

50	Sieboldianoside A or isomer	$C_{64}H_{104}O_{30}$	-0.03	1352.6607	1351.6528	16.05	$[M-H]^-$	1351, 881, 749, 603, 585, 471	12707777
51	Corosolic acid or isomer	$C_{30}H_{44}O_2$	0.19	436.3336	437.3415	16.06	$[M+H]^+$	439, 391, 247, 201, 191, 189, 159, 119	8836220
52	Oleanonic acid or isomer	$C_{30}H_{46}O_3$	-0.29	454.3441	455.3518	16.06	$[M+H]^+$	455, 437, 409, 391, 247, 201, 191, 119	20216133

53	(6,6-Dimethylbicyclo[3.1.1]hept-2-yl)methyl 6-O-[(2R,3R,4R)-3,4-dihydroxy-4-(hydroxymethyl)tetrahydro-2-furanyl]-β-D-glucopyranoside	$C_{21}H_{36}O_{10}$	1.47	448.2315	493.2297	16.07	$[M+FA-H]^-$	447, 315, 161,	174065490
54	21β-hydroxyoleanolic acid/21α-hydroxyoleanolic acid	$C_{30}H_{46}O_4$	-0.31	470.3391	471.3467	16.24	$[M+H]^+$	471, 425, 271, 235, 217, 189	12461599
55	18-β-Glycyrrhetinic acid	$C_{30}H_{46}O_4$	1.05	470.3401	471.3474	16.25	$[M+H]^+$	471, 425	12461599
56	Corosolic acid or isomer	$C_{30}H_{44}O_2$	-0.73	436.3336	437.3411	16.51	$[M+H]^+$	439, 391, 247, 201, 191, 189, 159, 119	74341170

57	Oleanonic acid or isomer	$C_{30}H_{46}O_3$	-0.84	454.344<br>1	455.35<br>16	16.<br>51	[M+H]<br>+	455,<br>437,<br>409,<br>391,<br>247,<br>201,<br>191,<br>119	713480<br>83
58	Androseptoside A	$C_{36}H_{58}O_8$	0.02	618.412<br>6	619.42<br>04	16.<br>51	[M+H]<br>+	619,<br>455,<br>437,<br>409,24<br>7, 201,<br>191,<br>119	343818<br>1
59	Corosolic acid or isomer	$C_{30}H_{44}O_2$	-0.73	436.333<br>6	437.34<br>11	16.<br>72	[M+H]<br>+	439,<br>391,<br>247,<br>201,<br>191,<br>189,<br>159,	178929<br>00

								119	
60	Oleanonic acid or isomer	$C_{30}H_{46}O_3$	-0.42	454.3441	455.3518	16.72	$[M+H]^+$	455, 437, 409, 391, 247, 201, 191, 119	17672095
61	Corosolic acid or isomer	$C_{30}H_{44}O_2$	-1	436.3336	437.341	16.93	$[M+H]^+$	439, 391, 247, 201, 191, 189, 159, 119	13430001
62	Oleanonic acid or isomer	$C_{30}H_{46}O_3$	-0.29	454.3441	455.3518	16.93	$[M+H]^+$	455, 437, 409, 391,	12173102

								247, 201, 191, 119	
63	21β-hydroxyoleanolic acid/21α-hydroxyoleanolic acid	$C_{30}H_{46}O_4$	1.05	470.3391	471.3474	17.67	$[M+H]^+$	471, 425, 271, 235, 217, 189	3650417
64	Corosolic acid or isomer	$C_{30}H_{44}O_2$	0.19	436.3336	437.3415	17.70	$[M+H]^+$	439, 391, 247, 201, 191, 189, 159, 119	17903788
65	Oleanonic acid or isomer	$C_{30}H_{46}O_3$	-0.84	454.3441	455.3516	17.70	$[M+H]^+$	455, 437, 409, 391,	24445676

								247, 201, 191, 119	
66	Sieboldianoside A or isomer	$C_{64}H_{104}O_{30}$	-0.03	1352.6607	1351.6528	17.71	$[M-H]^-$	1351, 881, 749, 603, 585, 471	13917220
67	3,4-Dicaffeoylquinic acid	$C_{25}H_{24}O_{12}$	1.55	516.1276	515.1203	18.53	$[M-H]^-$	515, 353 191, 179, 173, 135	31406819
68	Olean-12-en-28-oic acid or isomer	$C_{30}H_{46}O_2$	-1.81	438.3492	439.3563	18.82	$[M+H]^+$	439, 393, 249, 203, 191	552856197

69	Ursolic acid	$C_{30}H_{48}O_3$	-1.95	456.3595	457.3667	18.83	$[M+H]^+$	439, 411	32087646
70	Sieboldianoside B or isomer	$C_{64}H_{104}O_{29}$	1.19	1336.6658	1335.6595	18.86	$[M-H]^-$	865, 733, 587, 569, 455	8085227
71	Olean-12-en-28-oic acid or isomer	$C_{30}H_{46}O_2$	-1.47	438.3492	439.3564	19.02	$[M+H]^+$	439, 393, 249, 203, 191	80532441
72	Olean-12-en-28-oic acid or isomer	$C_{30}H_{46}O_2$	-2.16	438.3492	439.3561	19.27	$[M+H]^+$	439, 393, 249, 203, 191	150965203
73	Clematoside A	$C_{52}H_{84}O_{20}$	-2.85	1028.555	1027.5502	19.29	$[M-H]^-$	1027, 733, 587,	6979483

								455	
74	Skimmin	$C_{15}H_{16}O_8$	-0.93	324.0842	325.0915	19.71	$[M+H]^+$	325, 163, 145, 135	46617359
75	Olean-12-en-28-oic acid or isomer	$C_{30}H_{46}O_2$	-1.47	438.3492	439.3564	20.38	$[M+H]^+$	439, 393, 249, 203, 191	161899638
76	Sieboldianoside B or isomer	$C_{64}H_{104}O_{29}$	1.19	1336.6658	1335.6595	20.39	$[M-H]^-$	865, 733, 587, 569, 455	91026735
77	Sieboldianoside B or isomer	$C_{64}H_{104}O_{29}$	1.19	1336.6658	1335.6595	27.52	$[M-H]^-$	865, 733, 587, 569, 455	1230256

Supplementary Table S4.MRM transitions and calibration curves for quantitative detection.

Compound	MRM Transition (m/z)	Polarity	RT (min)	Calibration Equation	R^2
Magnoflorine	343.42→298.05	Pos	4.081	Y=445928X+647.2	0.9997
Phloretin	273.26→167.05	Neg	13.27	Y=541232X−16301	0.9909
Corosolic acid	471.35→471.35	Neg	15.456	Y=1174776X−43579	0.9972
Ursolic acid	455.4→455.4	Neg	16.62	Y=169107X−7644	0.9943
Oleanolic acid	455.35→455.35	Neg	16.511	Y=176297X−7914	0.9964
18β-Glycyrrhetinic acid	471.3→135	Pos	15.332	Y=292909X−15762	0.9941
Esculin	341.28→341.28	Pos	2.05	Y=135977X+5607	0.9970

Supplementary Note S5. Detailed Protocols for Molecular Docking and Molecular Dynamics Simulations

1. Molecular Docking Protocol

Molecular docking was performed using the Molecular Operating Environment (MOE) 2019.0102 platform.

Receptor Preparation: Five target proteins (PDB IDs: **PPARG: 9F7W, PTGS2: 5F19, PPARA: 6KAX, IL6: 4J4L, XDH: 2CKJ**) were retrieved from the RSCB PDB. The QuickPrep module was used to remove crystallographic water and ligands, add missing hydrogens (**Protonate3D**), and neutralize the system. Energy minimization was conducted with a convergence gradient of $0.1 \mathrm{kcal} \cdot \mathrm{mol}^{-1} \cdot \mathrm{Å}^{-1}$.

Docking Settings:

Placement: Triangle Matcher;

Scoring: London dG (initial) and GBVI/WSA dG (final);

Refinement: Rigid Receptor;

Output: 30 optimal conformations per ligand.

2. Molecular Dynamics (MD) Simulation Parameters

MD simulations were executed using **GROMACS 2023.2** to evaluate protein–ligand complex stability.

Force Field and Solvation: The **CHARMM36-jul2022** force field was applied. The complex was immersed in a cubic box of **SPC water** with a $1.0 \mathrm{nm}$ buffer. The system was neutralized with Cl^- or Na^+ ions.

Equilibration:
a. **Energy Minimization:** Steepest descent algorithm.
b. **NVT:** $100 \mathrm{ps}$ at $300 \mathrm{K}$ using the V-rescale thermostat (τ_t=0.1ps).
c. **NPT:** $100 \mathrm{ps}$ at $300 \mathrm{K}$ and $1.0 \mathrm{bar}$ using the Berendsen barostat (τ_p=2.0ps).

Production MD: A 50-ns production run used the Leap-frog integrator. Temperature (300K, V-rescale) and pressure (1.0bar, Parrinello–Rahman) were maintained.

Stability Analysis: System equilibrium was validated through Root Mean Square Deviation (RMSD), Root Mean Square Fluctuation (RMSF), and hydrogen bond evolution.

Supplementary Table S5. Sequences of Primers Used for RT-qPCR Analysis

Gene name	Forward Primer (5'-3')	Reverse Primer (5'-3')
oat1	tcccgctggattcctcctta	gcacaaccctctgtttgcag
hnf4a	taggggcaggcactctatgt	tggtttttgcgtacactgcg
hprt1	gaaggggcagagaacctgtc	ggcttgaaggcttcaacgtg
il-6	agcgtcttcaccgaagtctg	ccgattcagtctgaccggag
tnf-α	ctcgctgcagttgcttttgt	tttccgtggtctgaggaagc
il-1β	ctgaaatgatggcatgcggg	gctcattgcaagcggatctg
β-actin	cttccagccttccttcctgg	ctgcatacggtcagcaatgc
GAPDH	gtcaaggctgagaacgggaa	aaatgagccccagccttctc
PPARA	gcaatccatcggcgaggata	tgaaagcgtgtccgtgatga
PPARG	caactgcagatacatgccgc	tcggcagatctggactggta
IL6	agtgaggaacaagccagagc	ggtcaggggtggttattgca
IL1B	ccacctccagggacaggata	tcaacacgcaggacaggtac
URAT1	cgcttccggacctgtatctc	gggatgtccacgacaccaat
GLUT9	cctgctcttggagaagcaca	tcagccaggacctcctctac

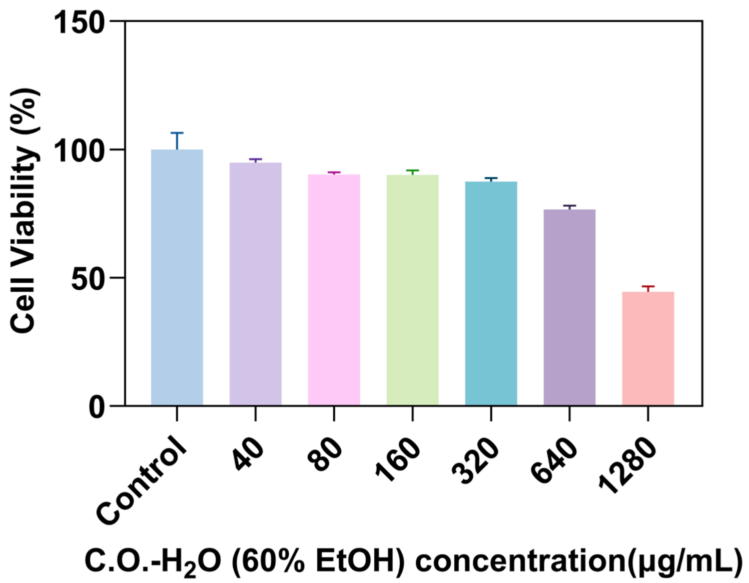

Supplementary Figure S4.Cross-validation of CWE-60EF cytotoxicity using the CCK-8 assay.HK-2 cells were treated with indicated concentrations of CWE-60EF for 24 hours. Cell viability was determined by the CCK-8 assay and is expressed as a percentage relative to the control group. The results confirm the lack of significant cytotoxicity at the tested concentrations. Data are presented as mean ± SD (n = 3).
